# Supplementary figures and images for: Combined administration of anisodamine and neostigmine alleviated colitis by inducing autophagy and inhibiting inflammation
Source: PLoS One. 2024 Feb 14;19(2):e0291543. doi: 10.1371/journal.pone.0291543 (PMC10866466; doi:10.1371/journal.pone.0291543)

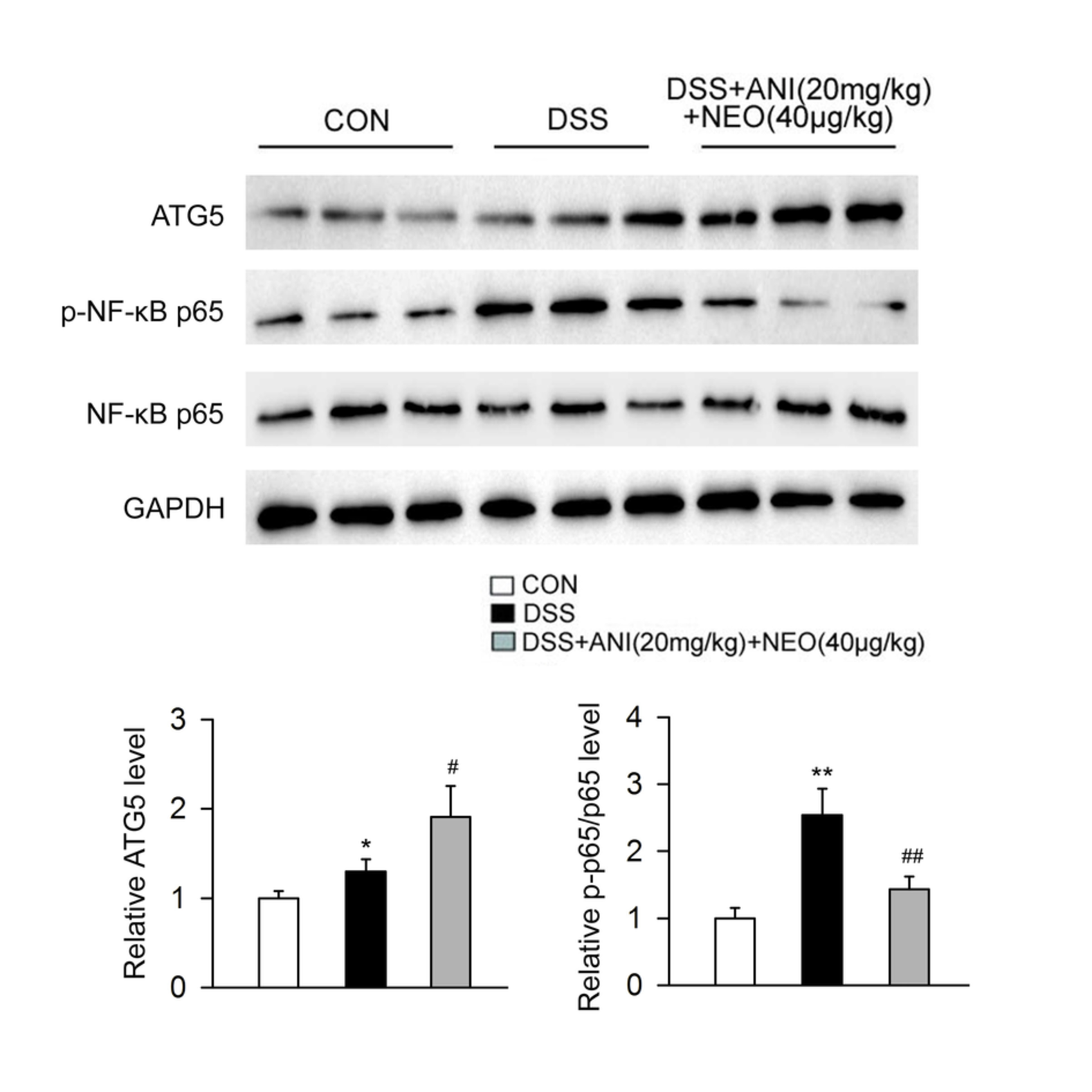

Supplement: S1 Fig — C57BL/6 mice were received 3% DSS for 7 days, the control mice were given tap water. Animals were treated with vehicle or ANI/NEO compound (20 mg/kg and 40 μg/kg, i.p.) twice a day from day 3 to day 7. The protein level of ATG5 and the phosphorylation level of NF-κB in colonic tissue were detected. Representative western blot and quantification data for ATG5 and p-NF-κB p65 in the intestinal tissues. n = 3 per group. *P<0.05 vs. the control group, **P<0.01 vs. the control group; #P<0.05 vs. the DSS group, ##P<0.01 vs. the DSS group. (TIF) [file pone.0291543.s001.tif]

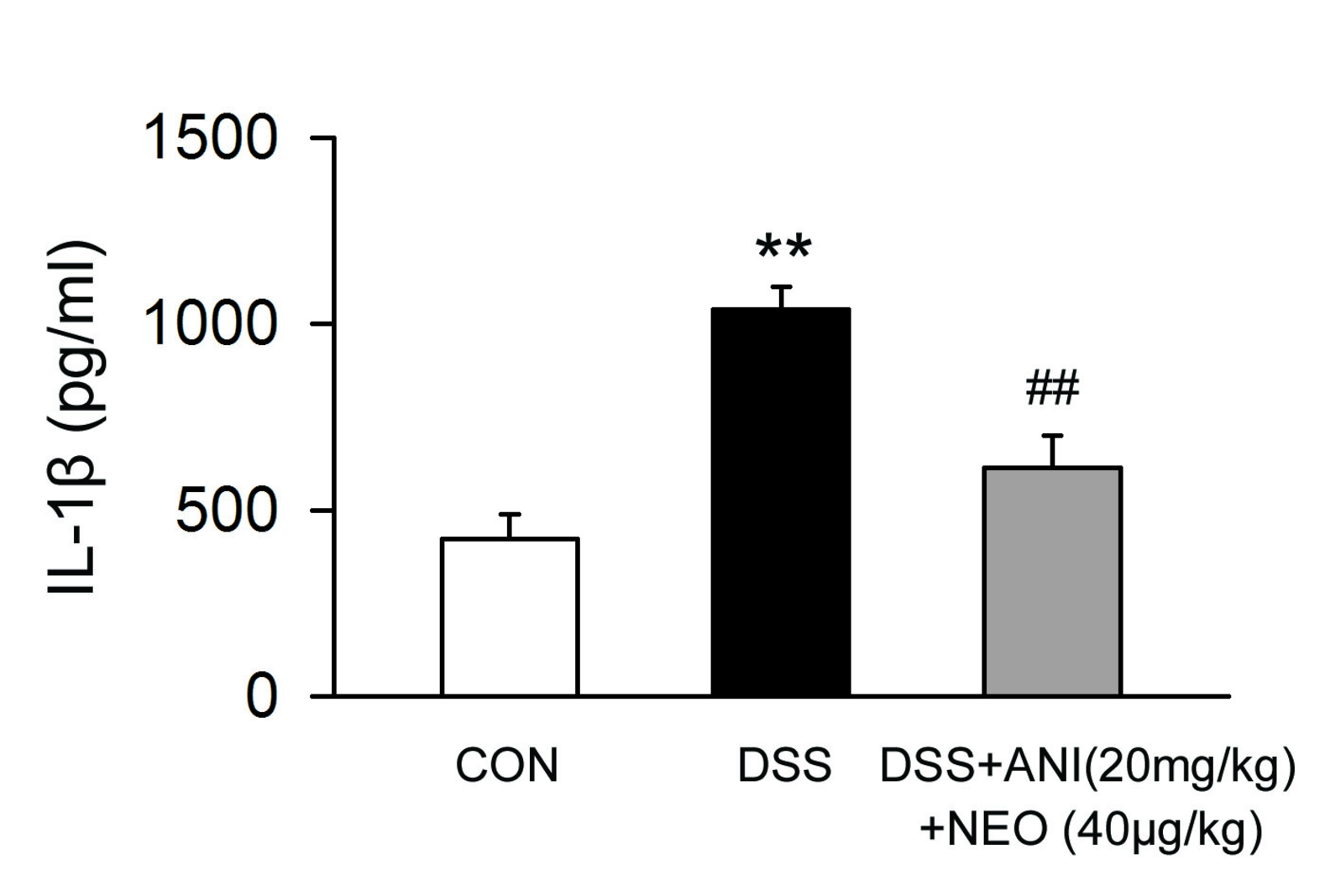

Supplement: S2 Fig — C57BL/6 mice were received 3% DSS for 7 days, the control mice were given tap water. Animals were treated with vehicle or ANI/NEO compound (20 mg/kg and 40 μg/kg, i.p.) twice a day from day 3 to day 7. The concentration of IL-1β in colonic tissue were detected. n = 4 per group. **P<0.01 vs. the control group; ##P<0.01 vs. the DSS group. (TIF) [file pone.0291543.s002.tif]

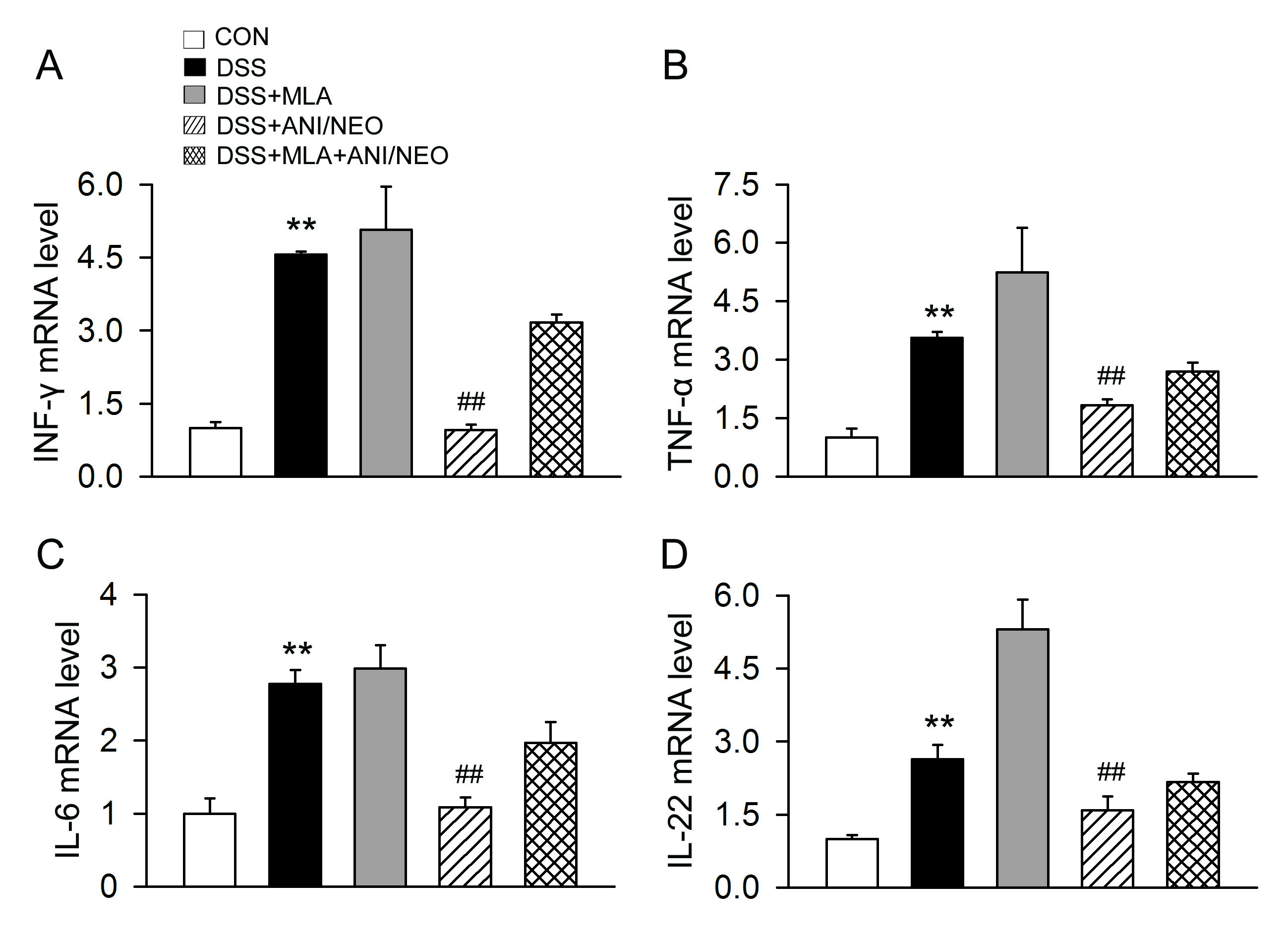

Supplement: S3 Fig — C57BL/6 mice were received 3% DSS for 7 days, the control mice were given tap water. The DSS mice were treated with vehicle, MLA (10 mg/kg, i.p.), ANI/NEO compound (20 mg/kg and 40 μg/kg, i.p.) or MLA+ ANI/NEO compound. MLA was given daily and ANI/NEO compound twice a day from day 3 to day 7. The expression of INF-γ, TNF-α, IL-6 and IL-22 in colonic tissue were detected with RT-PCR. n = 3 or 4 per group. **P<0.01 vs. the control group; ##P<0.01 vs. the DSS group. (TIF) [file pone.0291543.s003.tif]

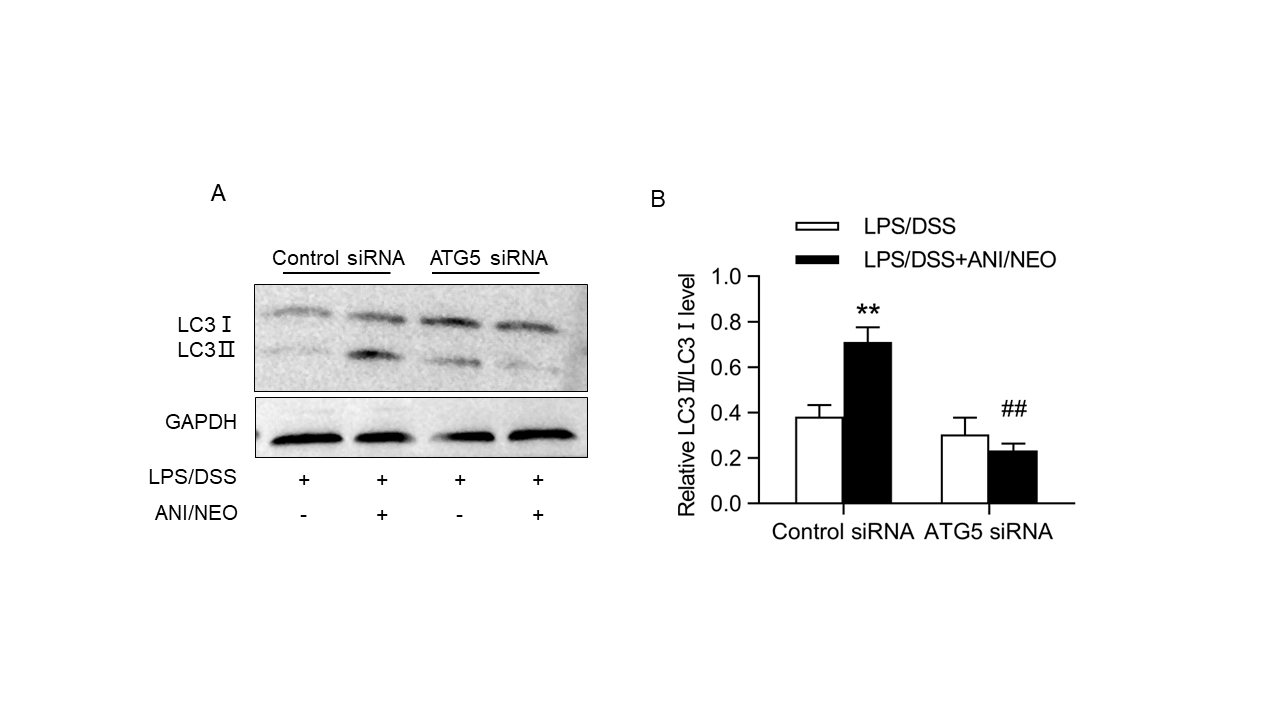

Supplement: S4 Fig — After transfection with control siRNA or ATG5 siRNA for 24h, Caco-2 cells were primed with 10 ng/ml LPS for 1 h, and then stimulated with 3% DSS in the presence or absence of 100 μM ANI/NEO compound for 24 h. (A-B) The LC3-II/LC3-I ratio in Caco-2 cells were analyzed. n = 3 per group. **P<0.01 vs. the Control siRNA LPS/DSS group; ##P<0.01 vs. the Control siRNA LPS/DSS+ANI/NEO group. (TIF) [file pone.0291543.s004.tif]

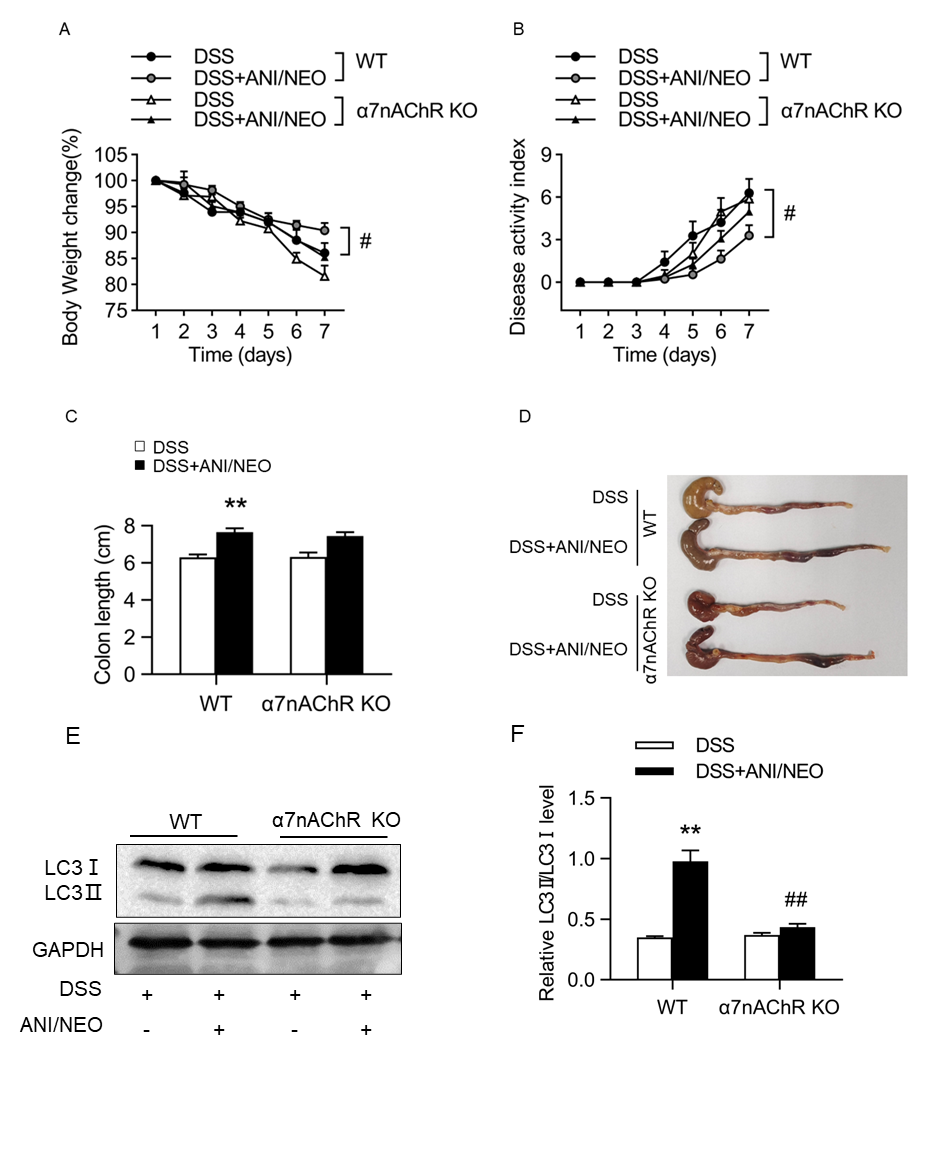

Supplement: S5 Fig — WT and α7nAChR KO mice were received 3% DSS for 7 days and the DSS mice were treated with vehicle or ANI/NEO compound (20 mg/kg and 40 μg/kg, i.p.) twice a day from day 3 to day 7. (A) The body weight loss was tested in the mice. n = 4 per group. #P<0.05 vs. the WT DSS group. (B) The disease activity index was tested in the mice. n = 4 per group. #P<0.05 vs. the WT DSS group. (C-D) The colon length was tested in the mice. n = 4 per group. **P<0.01 vs. the WT DSS group. (E-F) The LC3-II/LC3-I ratio in colonic tissues were analyzed. n = 3 per group. **P<0.01 vs. the WT DSS group; ##P<0.01 vs. the WT DSS+ANI/NEO group. (TIF) [file pone.0291543.s005.tif]

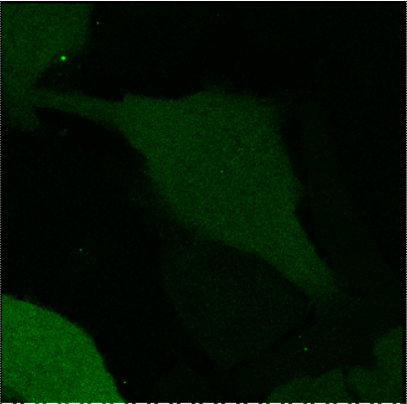

Supplement: S1 Data — (ZIP) [file pone.0291543.s006.zip › Autophagy Flux/CONTROL/con1-1.png]

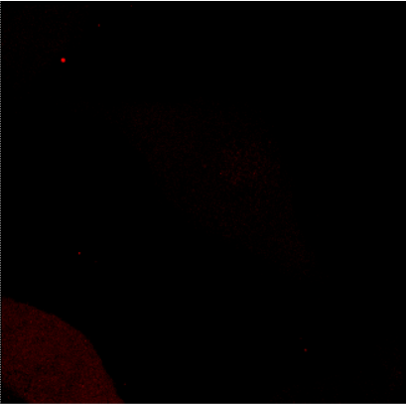

Supplement: S1 Data — (ZIP) [file pone.0291543.s006.zip › Autophagy Flux/CONTROL/con1-2.png]

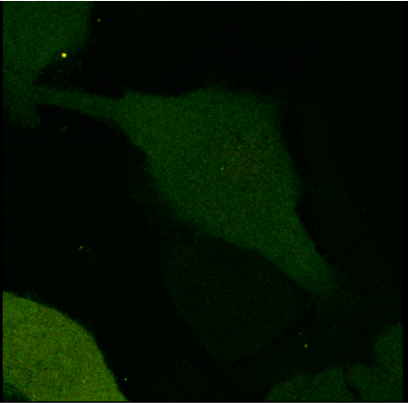

Supplement: S1 Data — (ZIP) [file pone.0291543.s006.zip › Autophagy Flux/CONTROL/con1-3.png]

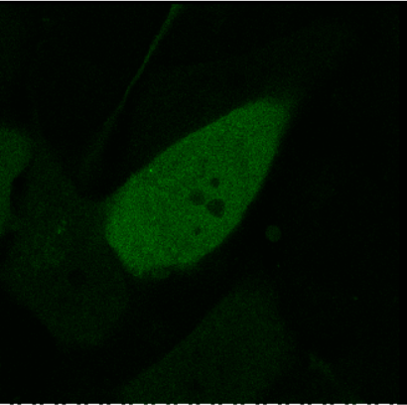

Supplement: S1 Data — (ZIP) [file pone.0291543.s006.zip › Autophagy Flux/CONTROL/con2-1.png]

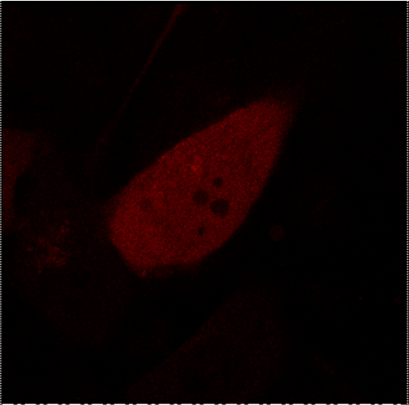

Supplement: S1 Data — (ZIP) [file pone.0291543.s006.zip › Autophagy Flux/CONTROL/con2-2.png]

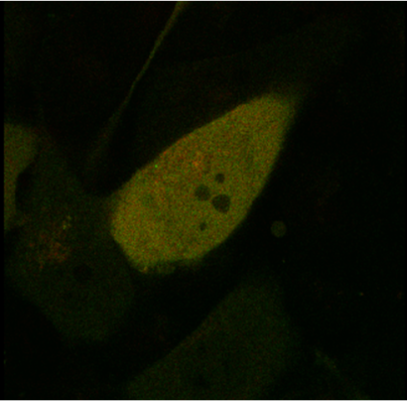

Supplement: S1 Data — (ZIP) [file pone.0291543.s006.zip › Autophagy Flux/CONTROL/con2-3.png]

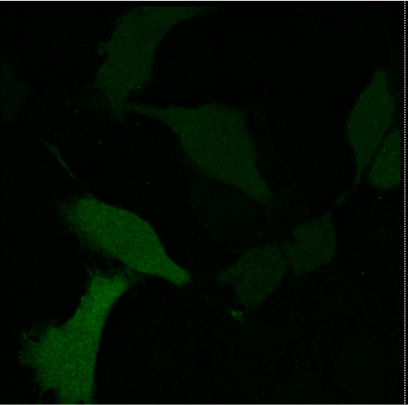

Supplement: S1 Data — (ZIP) [file pone.0291543.s006.zip › Autophagy Flux/CONTROL/con3-1.png]

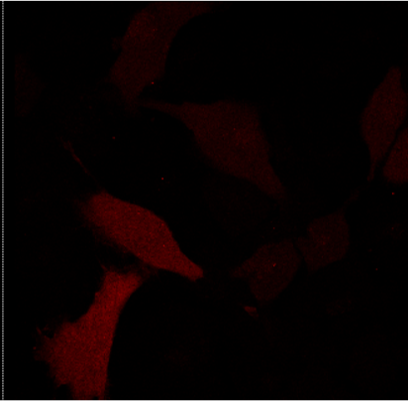

Supplement: S1 Data — (ZIP) [file pone.0291543.s006.zip › Autophagy Flux/CONTROL/con3-2.png]

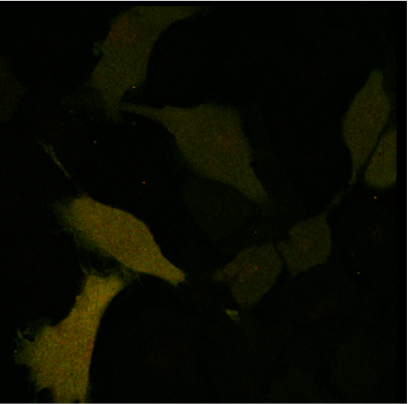

Supplement: S1 Data — (ZIP) [file pone.0291543.s006.zip › Autophagy Flux/CONTROL/con3-3.png]

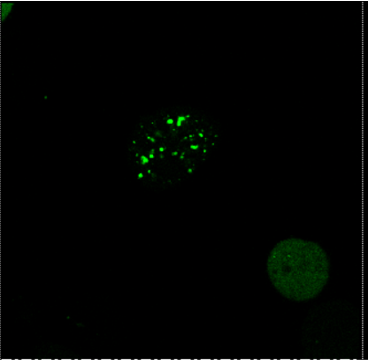

Supplement: S1 Data — (ZIP) [file pone.0291543.s006.zip › Autophagy Flux/LPSDSS/LPS.DSS1-1.png]

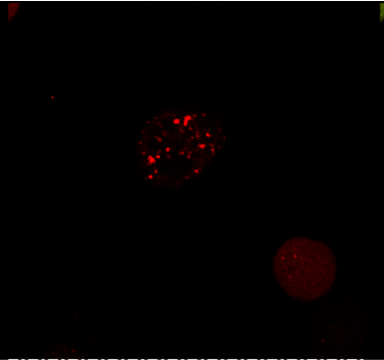

Supplement: S1 Data — (ZIP) [file pone.0291543.s006.zip › Autophagy Flux/LPSDSS/LPS.DSS1-2.png]

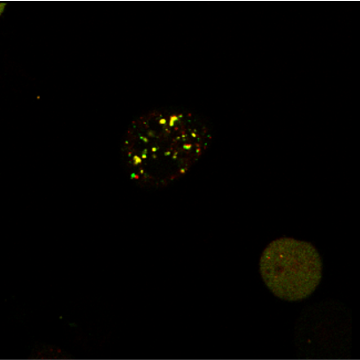

Supplement: S1 Data — (ZIP) [file pone.0291543.s006.zip › Autophagy Flux/LPSDSS/LPS.DSS1-3.png]

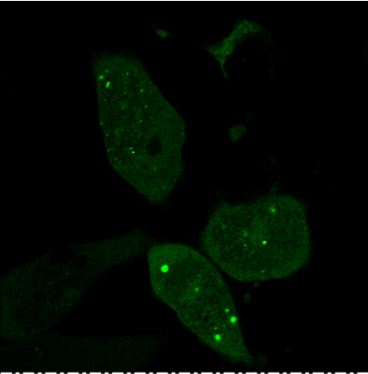

Supplement: S1 Data — (ZIP) [file pone.0291543.s006.zip › Autophagy Flux/LPSDSS/LPS.DSS2-1.png]

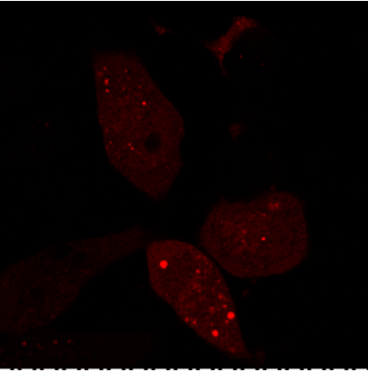

Supplement: S1 Data — (ZIP) [file pone.0291543.s006.zip › Autophagy Flux/LPSDSS/LPS.DSS2-2.png]

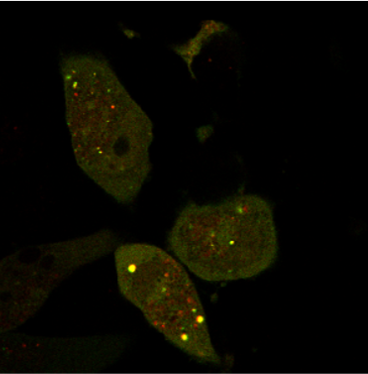

Supplement: S1 Data — (ZIP) [file pone.0291543.s006.zip › Autophagy Flux/LPSDSS/LPS.DSS2-3.png]

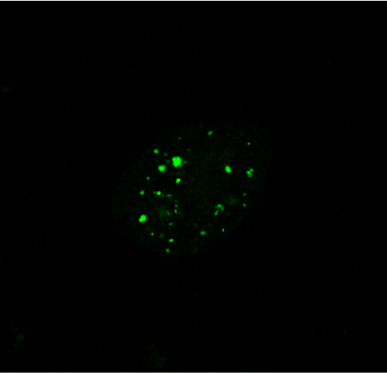

Supplement: S1 Data — (ZIP) [file pone.0291543.s006.zip › Autophagy Flux/LPSDSS/LPS.DSS3-1.png]

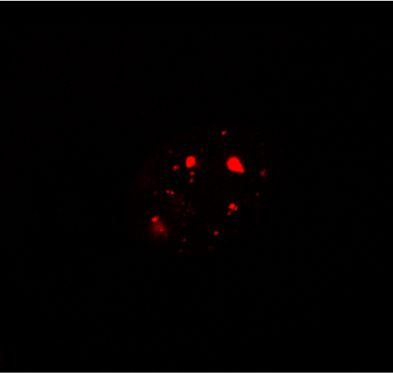

Supplement: S1 Data — (ZIP) [file pone.0291543.s006.zip › Autophagy Flux/LPSDSS/LPS.DSS3-2.png]

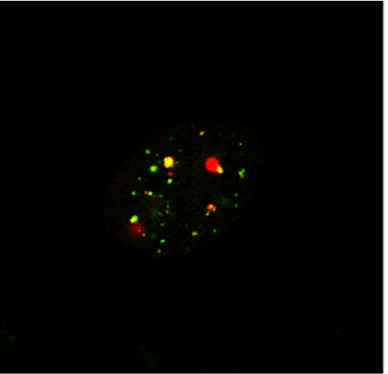

Supplement: S1 Data — (ZIP) [file pone.0291543.s006.zip › Autophagy Flux/LPSDSS/LPS.DSS3-3.png]

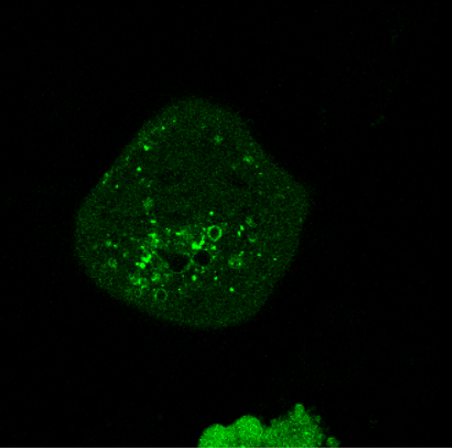

Supplement: S1 Data — (ZIP) [file pone.0291543.s006.zip › Autophagy Flux/LPSDSS+ANINEO/LPS.DSS+ANI NEO 1-1.png]

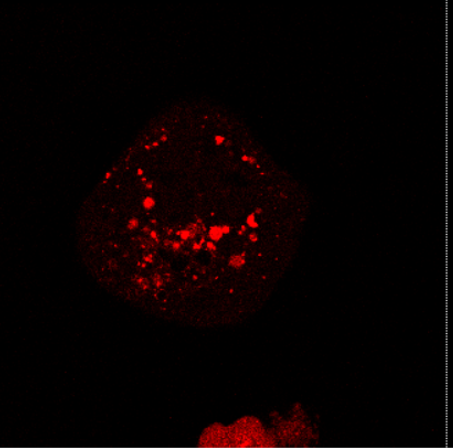

Supplement: S1 Data — (ZIP) [file pone.0291543.s006.zip › Autophagy Flux/LPSDSS+ANINEO/LPS.DSS+ANI NEO 1-2.png]

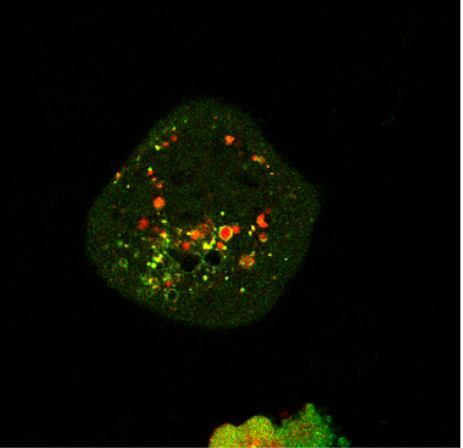

Supplement: S1 Data — (ZIP) [file pone.0291543.s006.zip › Autophagy Flux/LPSDSS+ANINEO/LPS.DSS+ANI NEO 1-3.png]

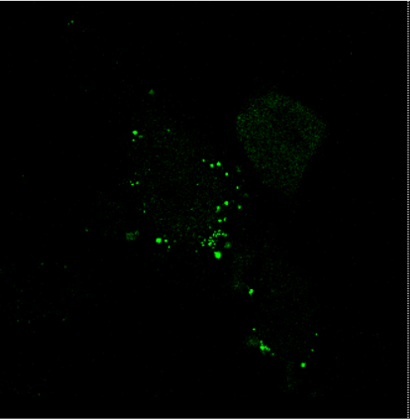

Supplement: S1 Data — (ZIP) [file pone.0291543.s006.zip › Autophagy Flux/LPSDSS+ANINEO/LPS.DSS+ANI NEO 2-1.png]

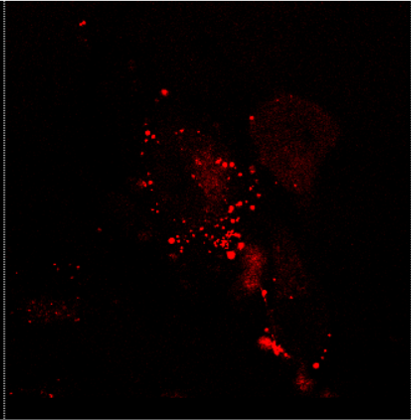

Supplement: S1 Data — (ZIP) [file pone.0291543.s006.zip › Autophagy Flux/LPSDSS+ANINEO/LPS.DSS+ANI NEO 2-2.png]

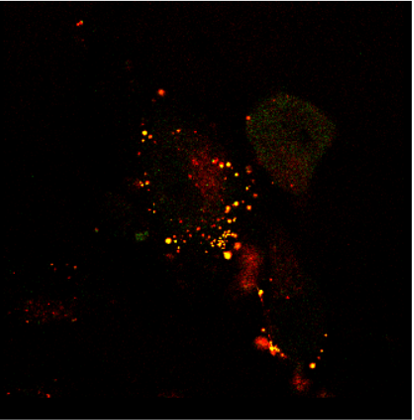

Supplement: S1 Data — (ZIP) [file pone.0291543.s006.zip › Autophagy Flux/LPSDSS+ANINEO/LPS.DSS+ANI NEO 2-3.png]

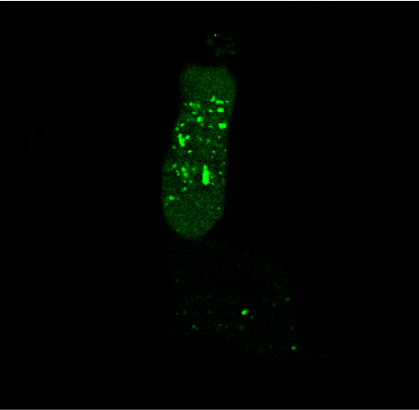

Supplement: S1 Data — (ZIP) [file pone.0291543.s006.zip › Autophagy Flux/LPSDSS+ANINEO/LPS.DSS+ANI NEO 3-1.png]

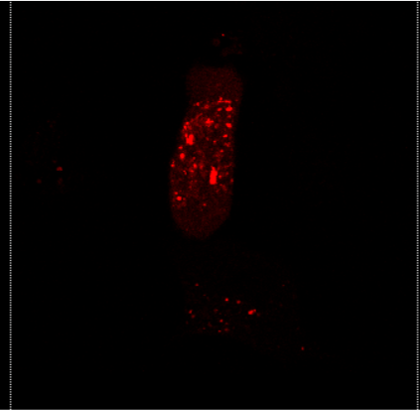

Supplement: S1 Data — (ZIP) [file pone.0291543.s006.zip › Autophagy Flux/LPSDSS+ANINEO/LPS.DSS+ANI NEO 3-2.png]

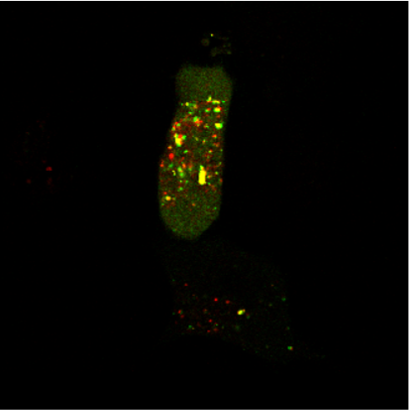

Supplement: S1 Data — (ZIP) [file pone.0291543.s006.zip › Autophagy Flux/LPSDSS+ANINEO/LPS.DSS+ANI NEO 3-3.png]

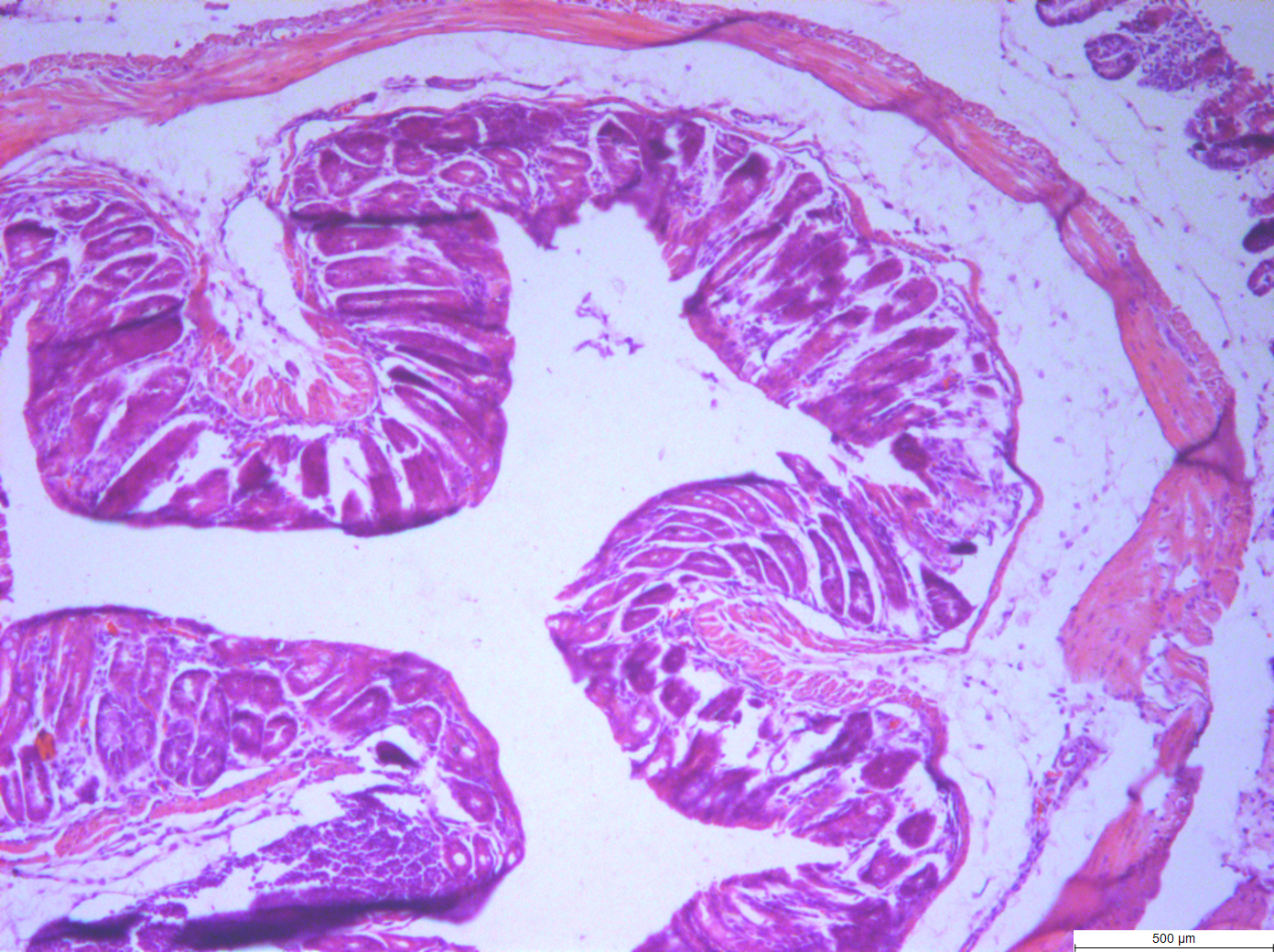

Supplement: S1 Data — (ZIP) [file pone.0291543.s006.zip › HE/CONTROL/CON 1.tif]

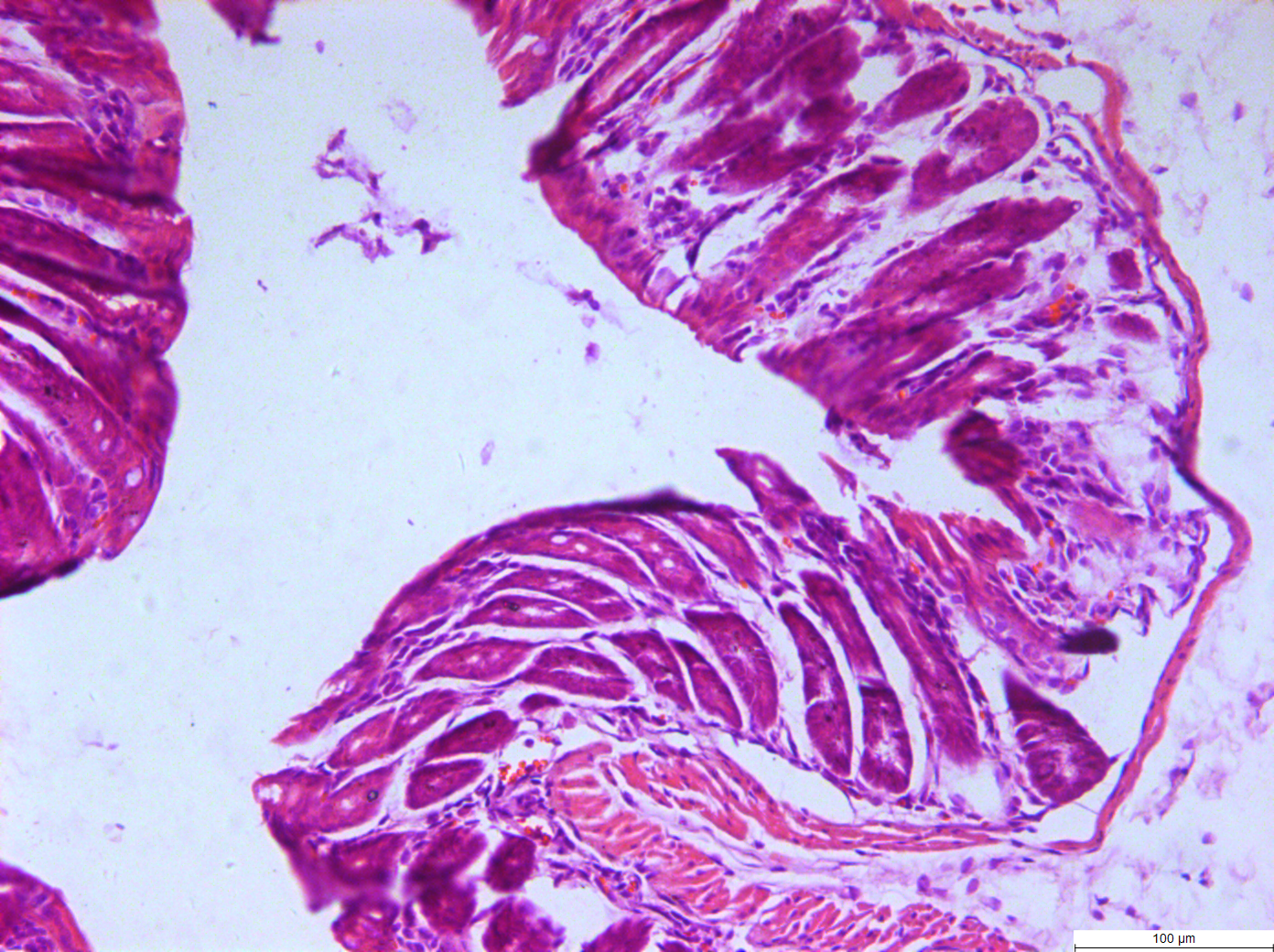

Supplement: S1 Data — (ZIP) [file pone.0291543.s006.zip › HE/CONTROL/CON2.tif]

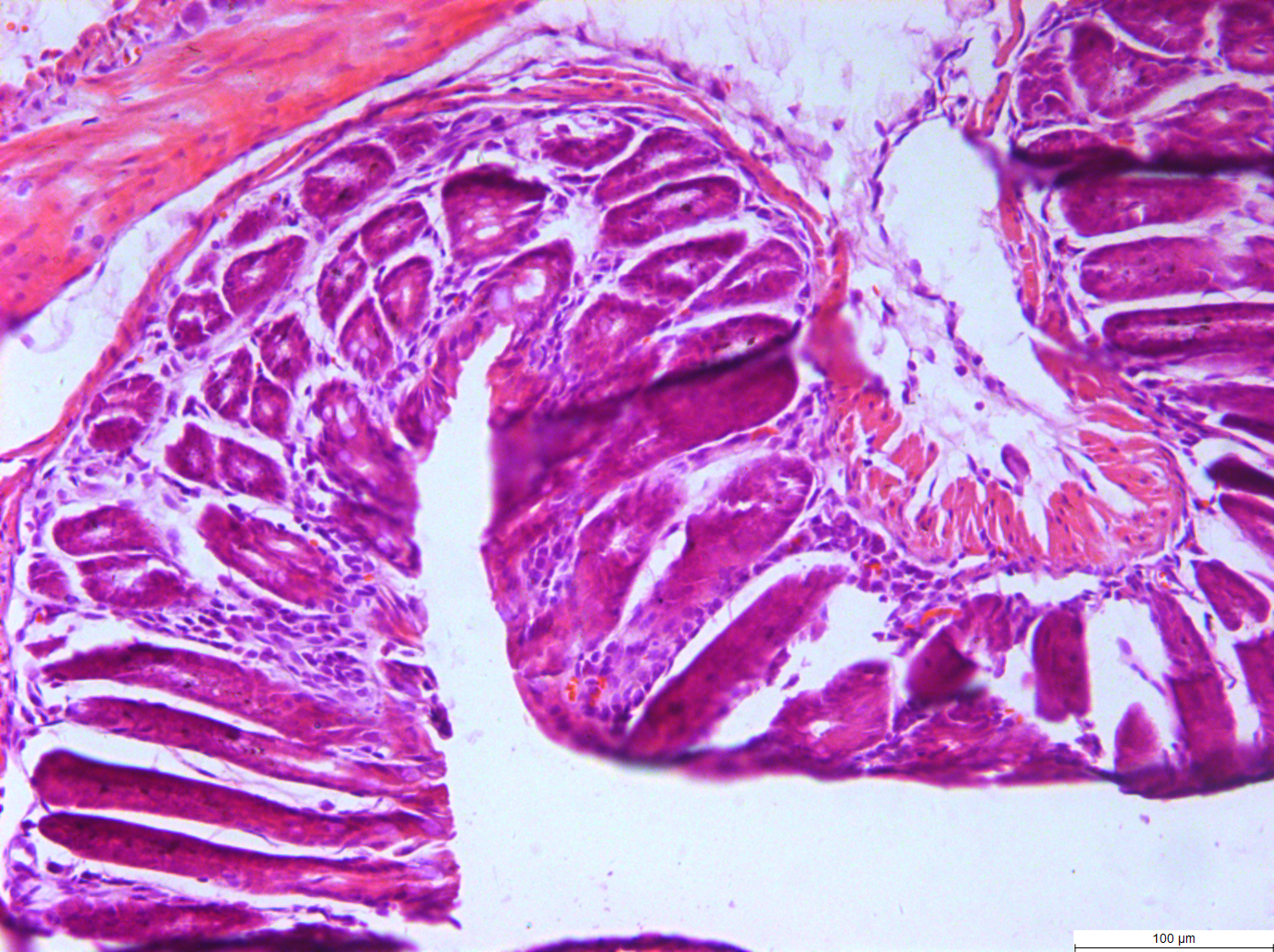

Supplement: S1 Data — (ZIP) [file pone.0291543.s006.zip › HE/CONTROL/CON3.tif]

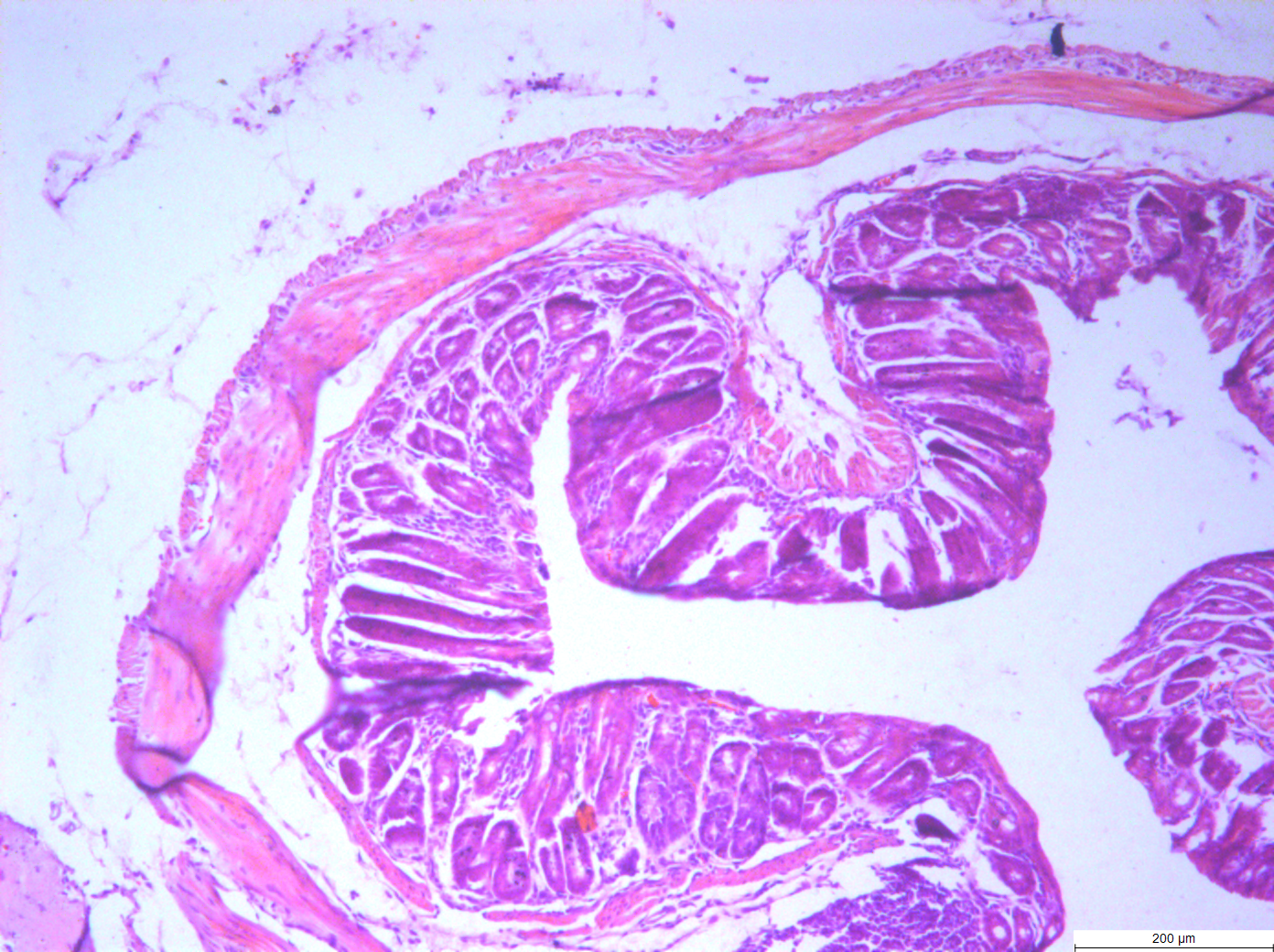

Supplement: S1 Data — (ZIP) [file pone.0291543.s006.zip › HE/CONTROL/CON4.tif]

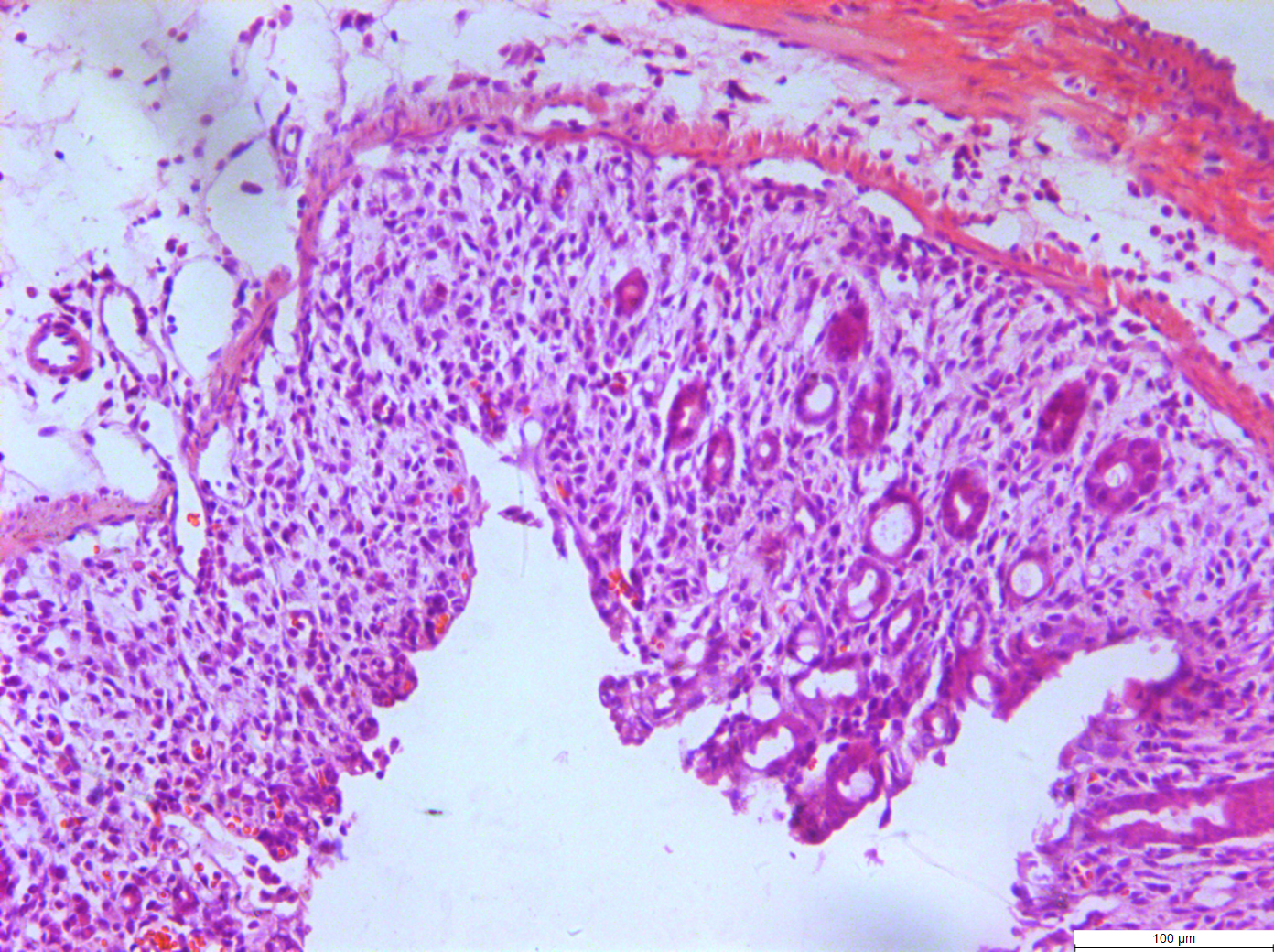

Supplement: S1 Data — (ZIP) [file pone.0291543.s006.zip › HE/DSS/DSS1.tif]

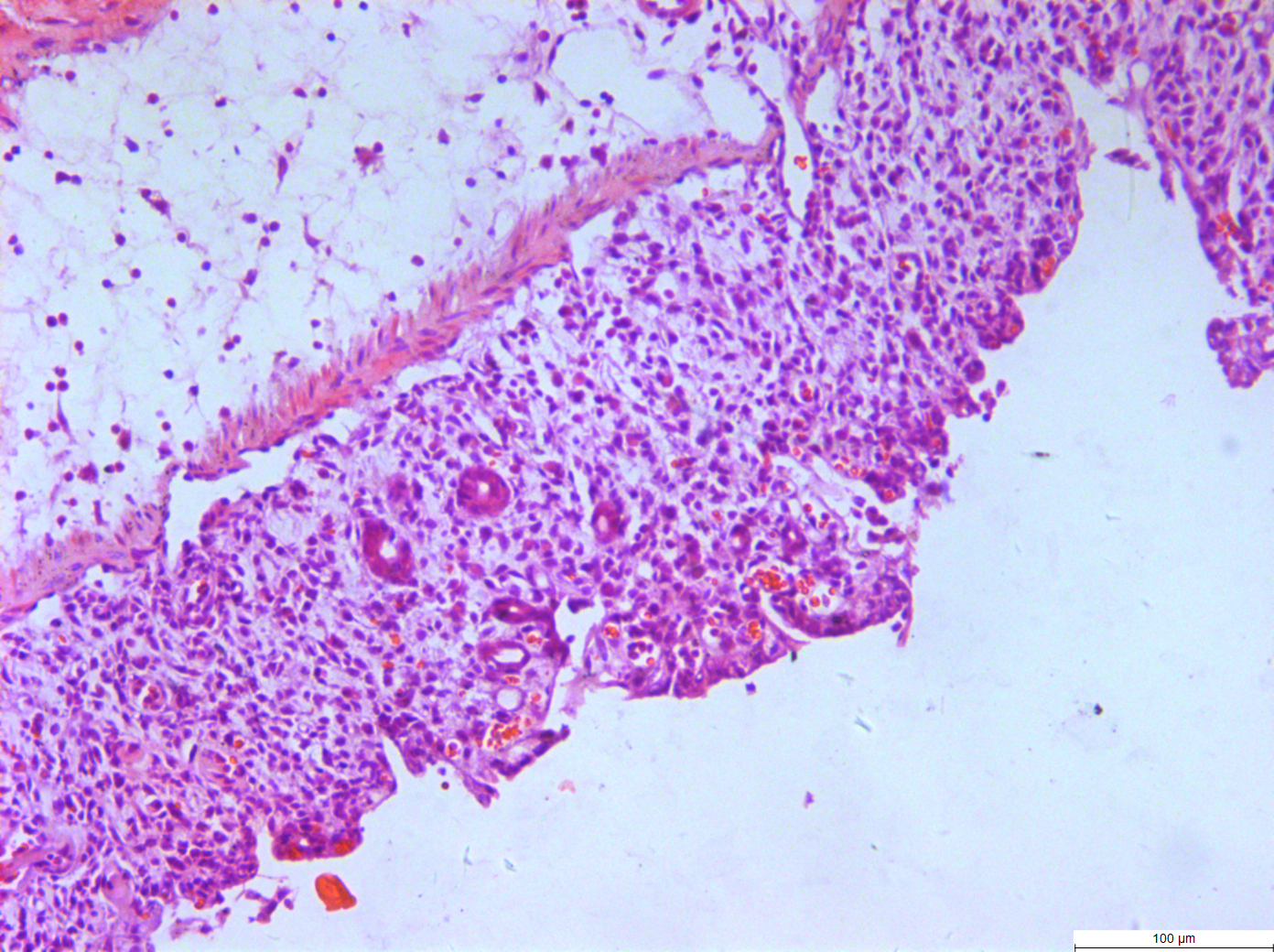

Supplement: S1 Data — (ZIP) [file pone.0291543.s006.zip › HE/DSS/DSS2.tif]

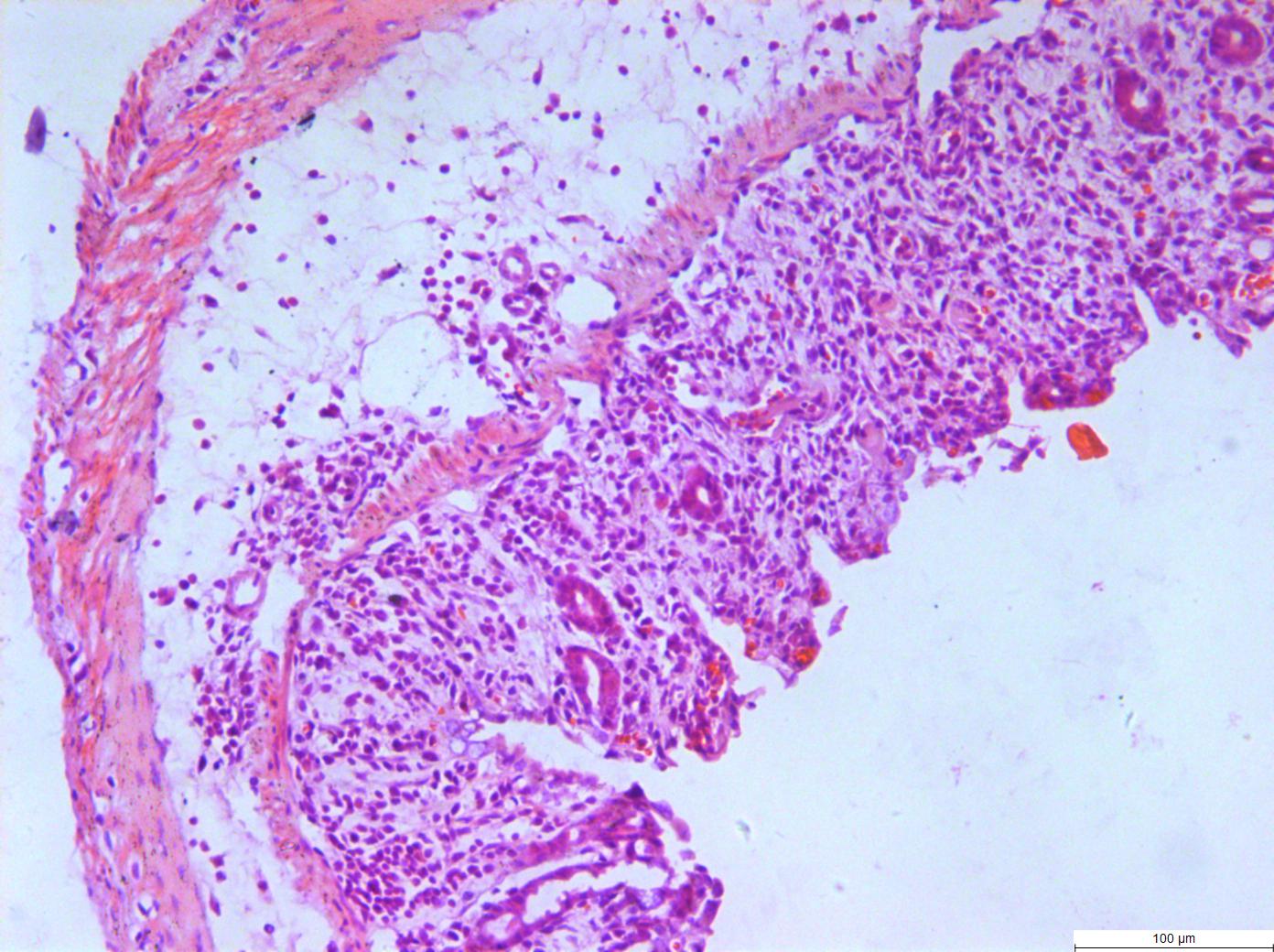

Supplement: S1 Data — (ZIP) [file pone.0291543.s006.zip › HE/DSS/DSS3.tif]

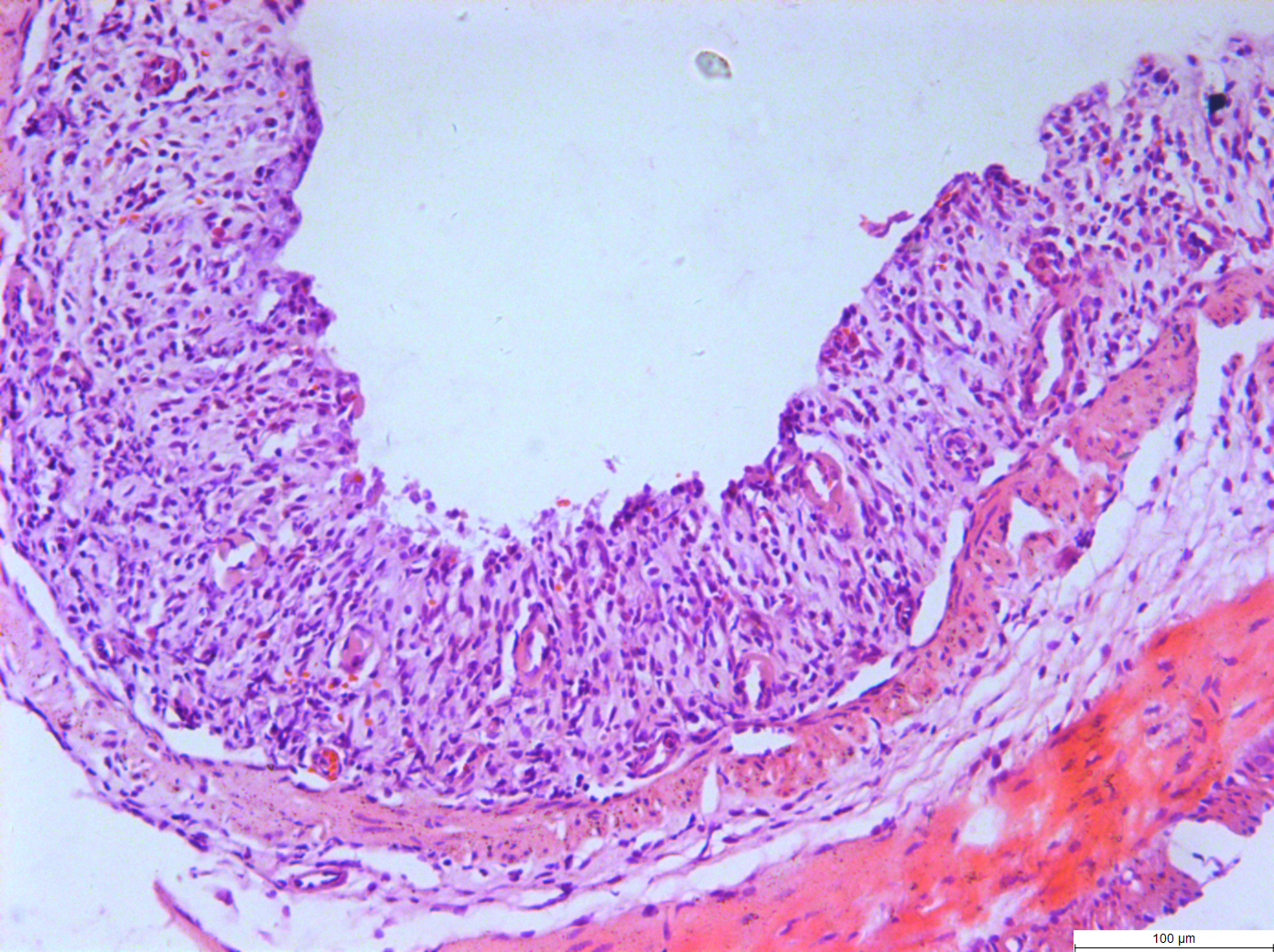

Supplement: S1 Data — (ZIP) [file pone.0291543.s006.zip › HE/DSS/DSS4.tif]

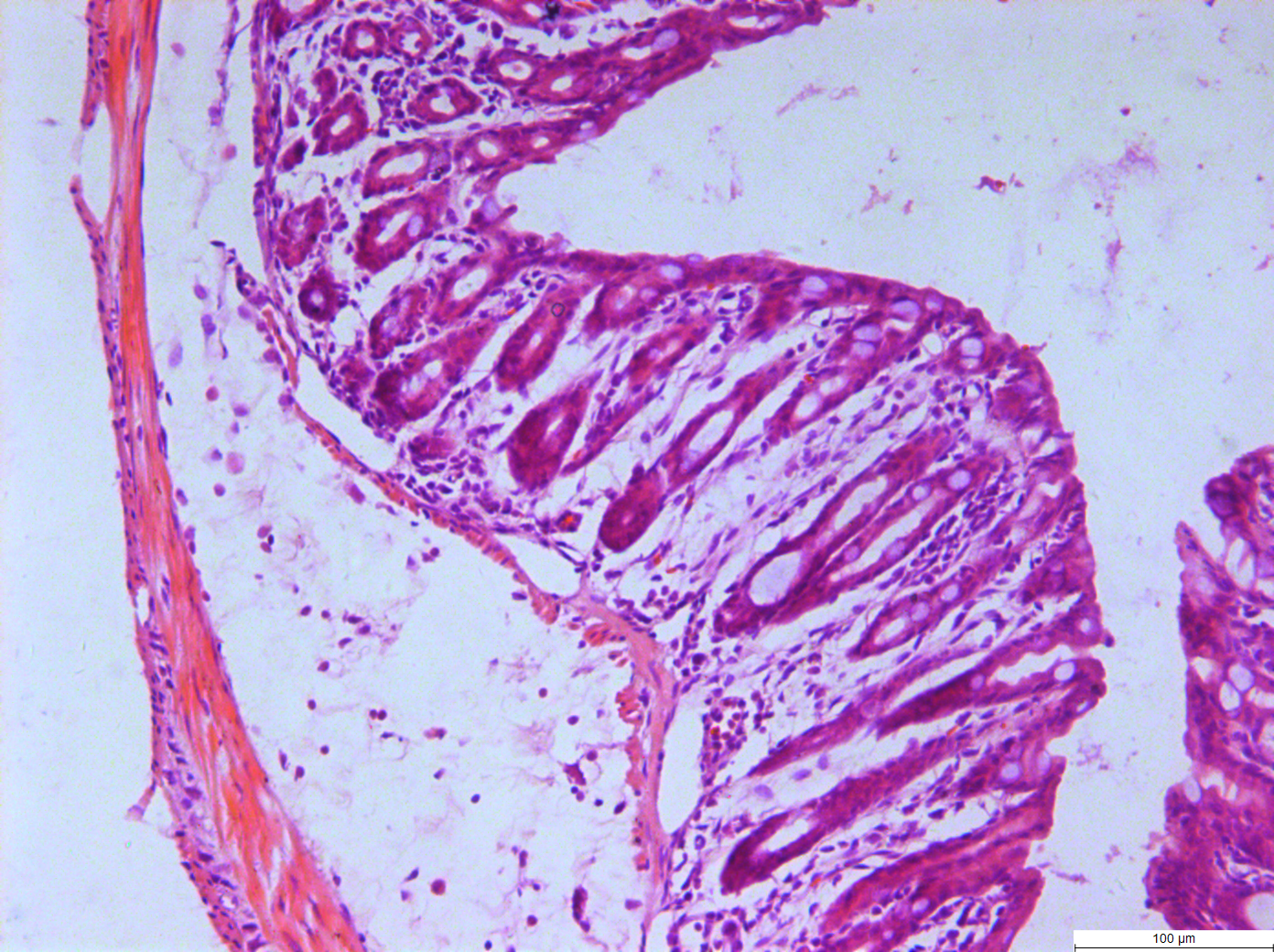

Supplement: S1 Data — (ZIP) [file pone.0291543.s006.zip › HE/DSS+ANINEO-10mg per kg/DSS+ANINEO-10mg per kg 1.tif]

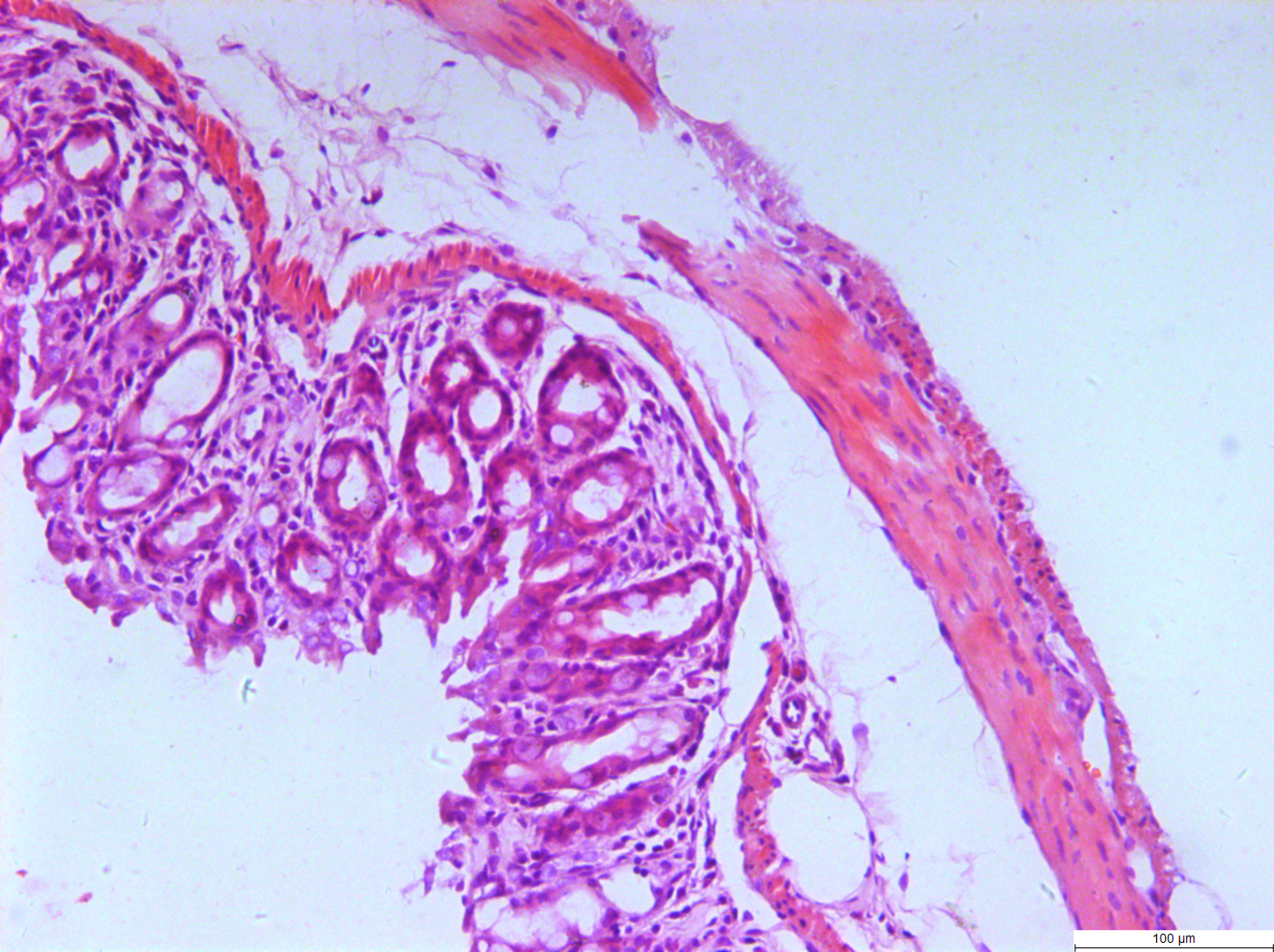

Supplement: S1 Data — (ZIP) [file pone.0291543.s006.zip › HE/DSS+ANINEO-10mg per kg/DSS+ANINEO-10mg per kg 2.tif]

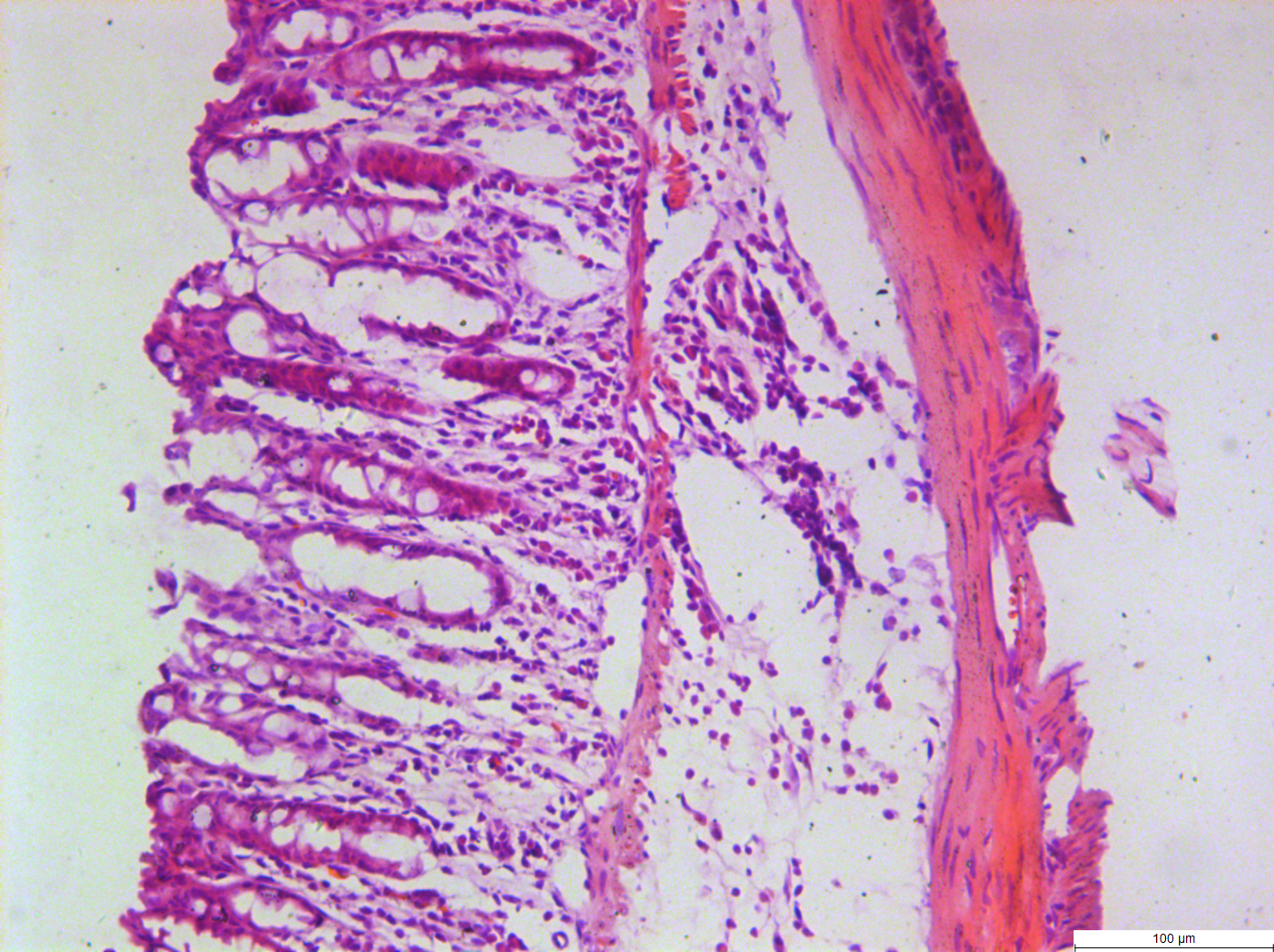

Supplement: S1 Data — (ZIP) [file pone.0291543.s006.zip › HE/DSS+ANINEO-10mg per kg/DSS+ANINEO-10mg per kg 3.tif]

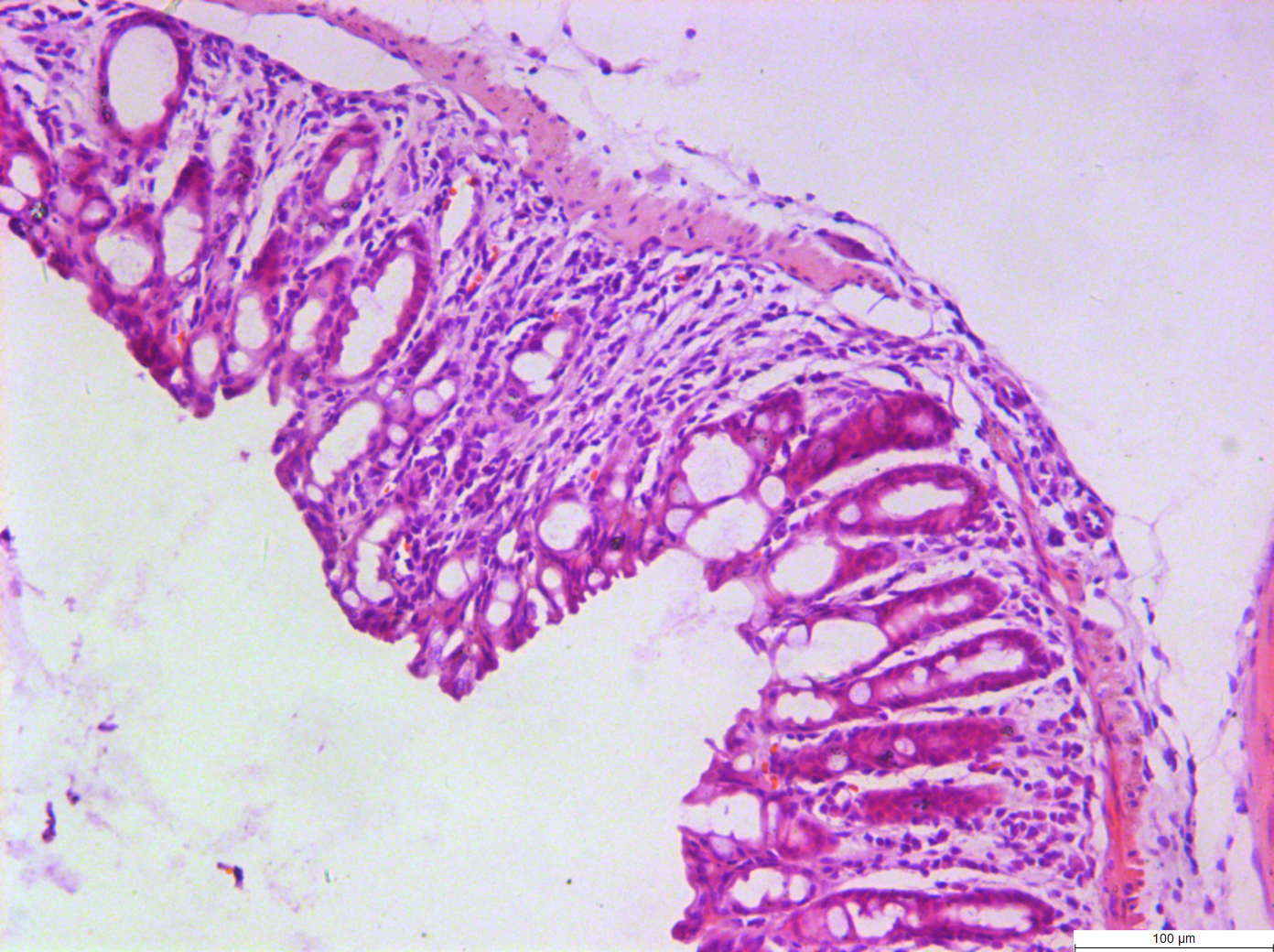

Supplement: S1 Data — (ZIP) [file pone.0291543.s006.zip › HE/DSS+ANINEO-10mg per kg/DSS+ANINEO-10mg per kg 4.tif]

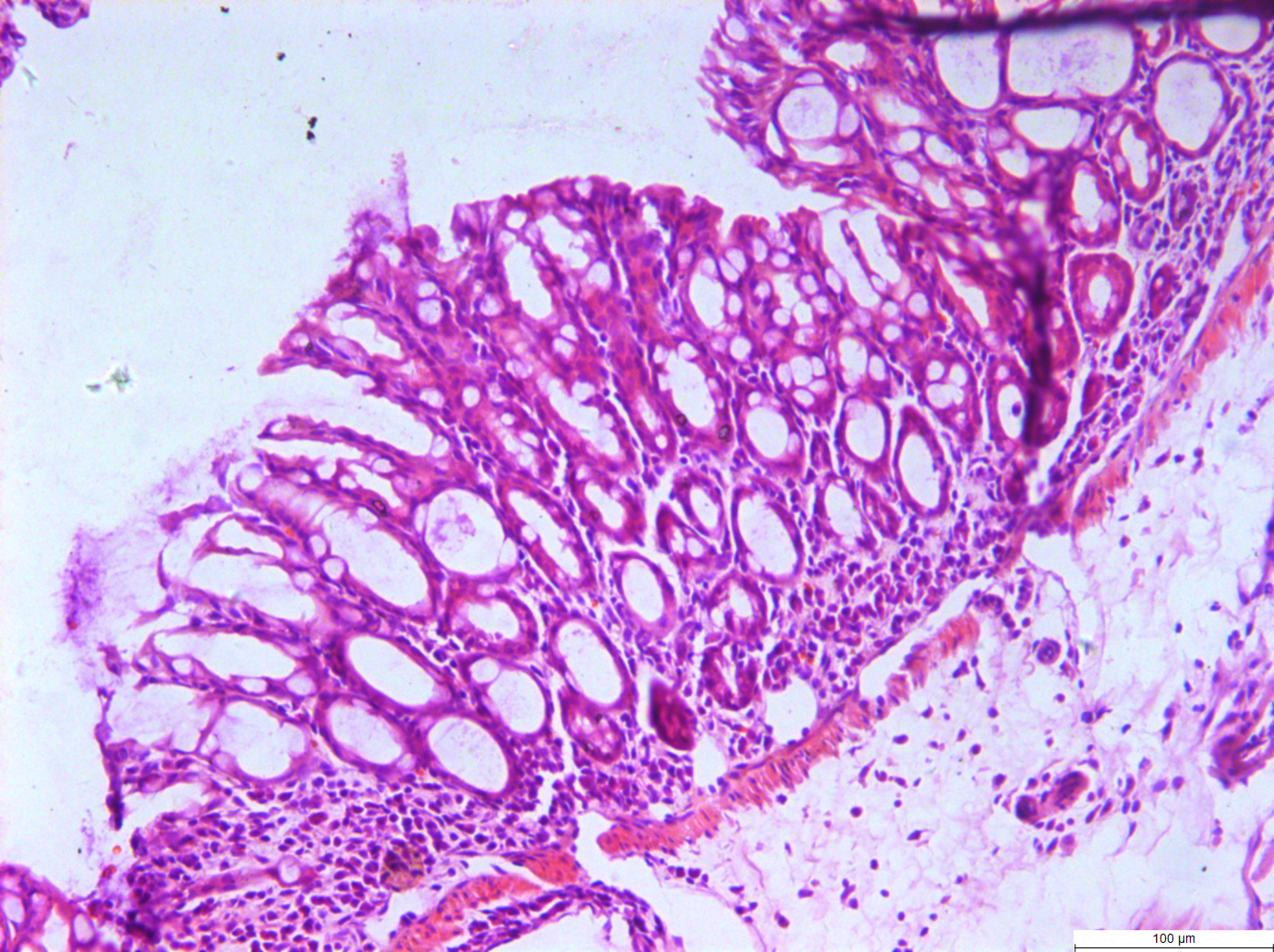

Supplement: S1 Data — (ZIP) [file pone.0291543.s006.zip › HE/DSS+ANINEO-20mg per kg/DSS+ANINEO-20mg per kg 1.tif]

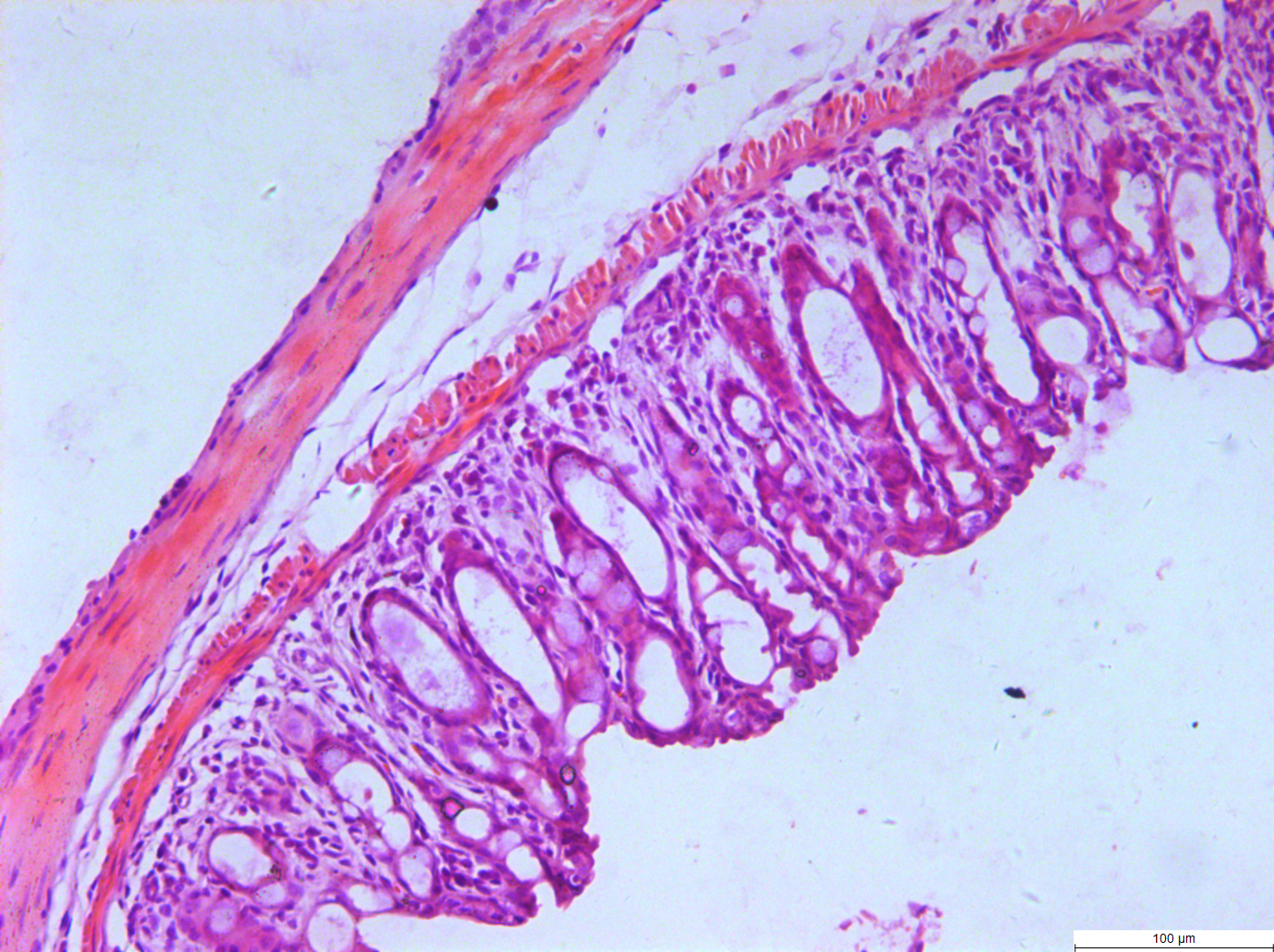

Supplement: S1 Data — (ZIP) [file pone.0291543.s006.zip › HE/DSS+ANINEO-20mg per kg/DSS+ANINEO-20mg per kg 2.tif]

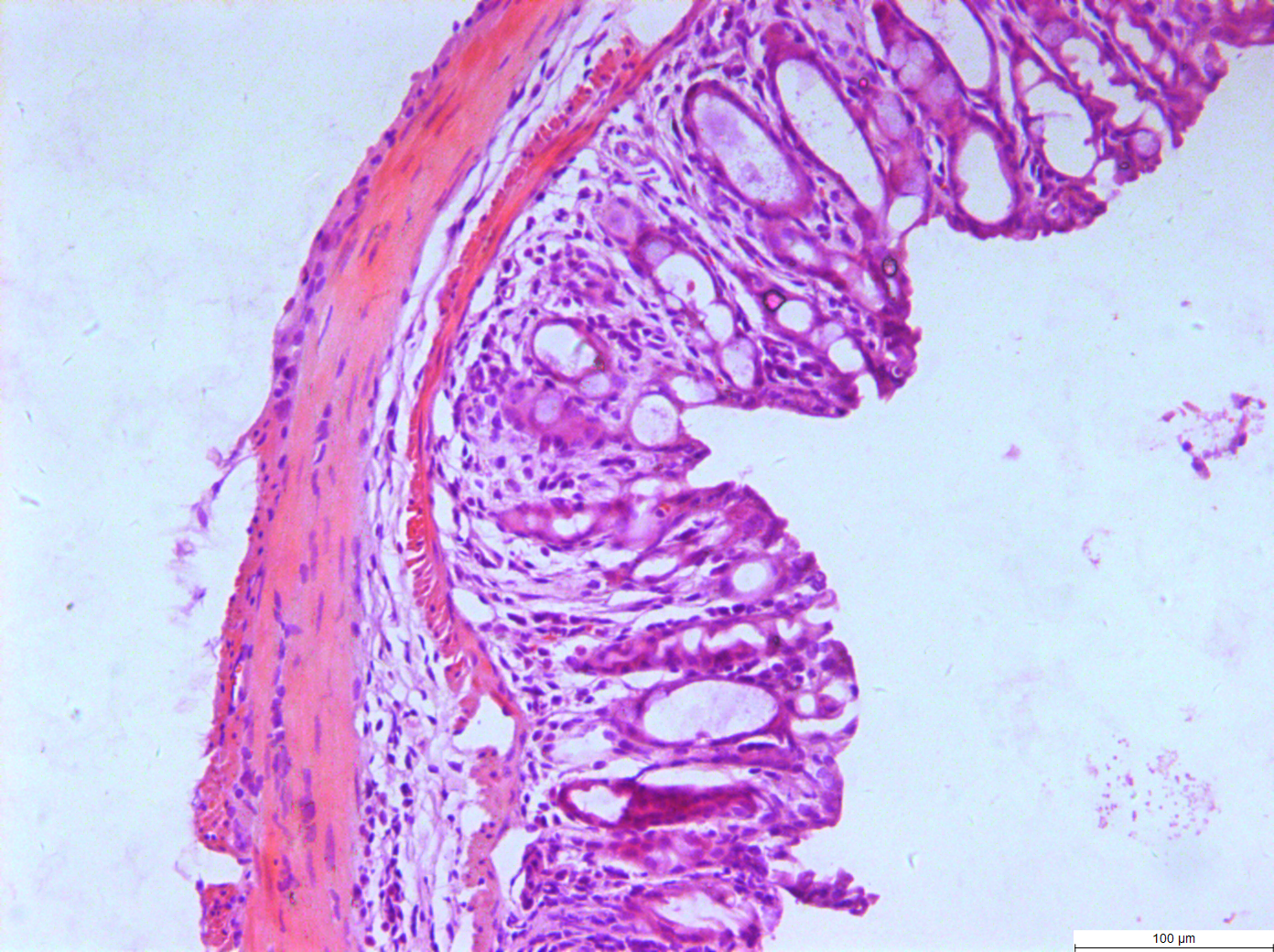

Supplement: S1 Data — (ZIP) [file pone.0291543.s006.zip › HE/DSS+ANINEO-20mg per kg/DSS+ANINEO-20mg per kg 3.tif]

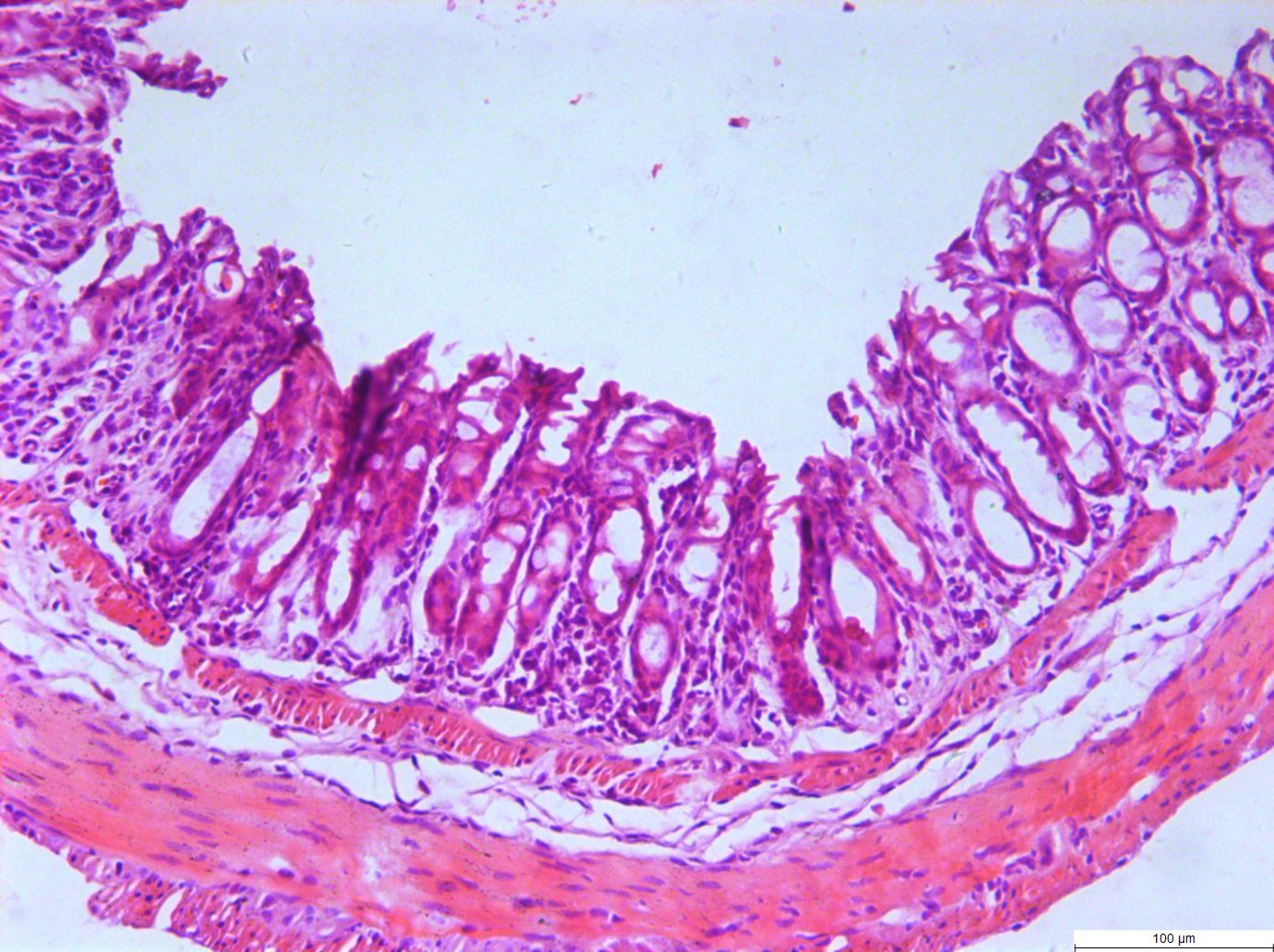

Supplement: S1 Data — (ZIP) [file pone.0291543.s006.zip › HE/DSS+ANINEO-20mg per kg/DSS+ANINEO-20mg per kg 4.tif]

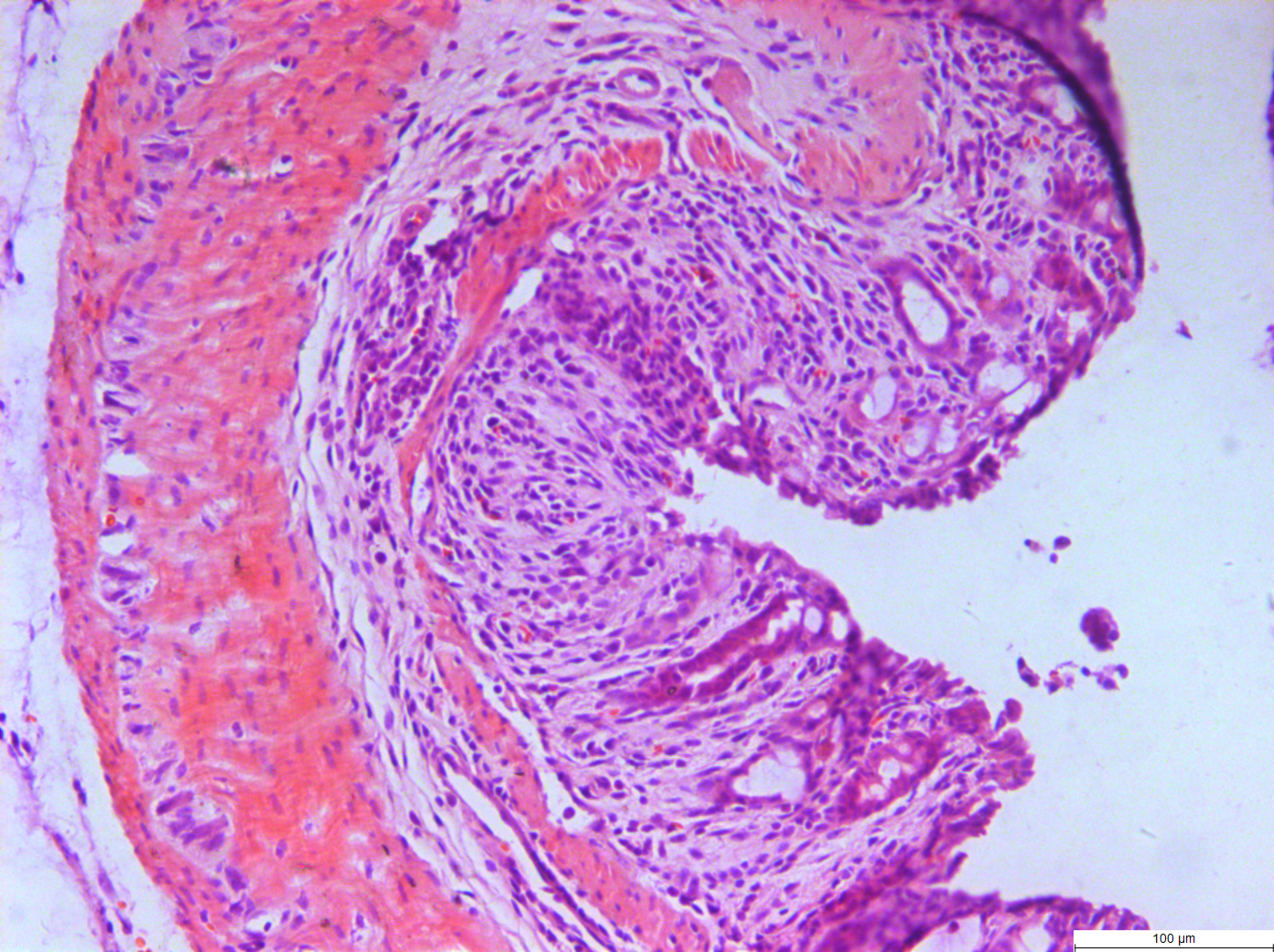

Supplement: S1 Data — (ZIP) [file pone.0291543.s006.zip › HE/DSS+ANINEO-5mg per kg/DSS+ANINEO-5mg per kg 1.tif]

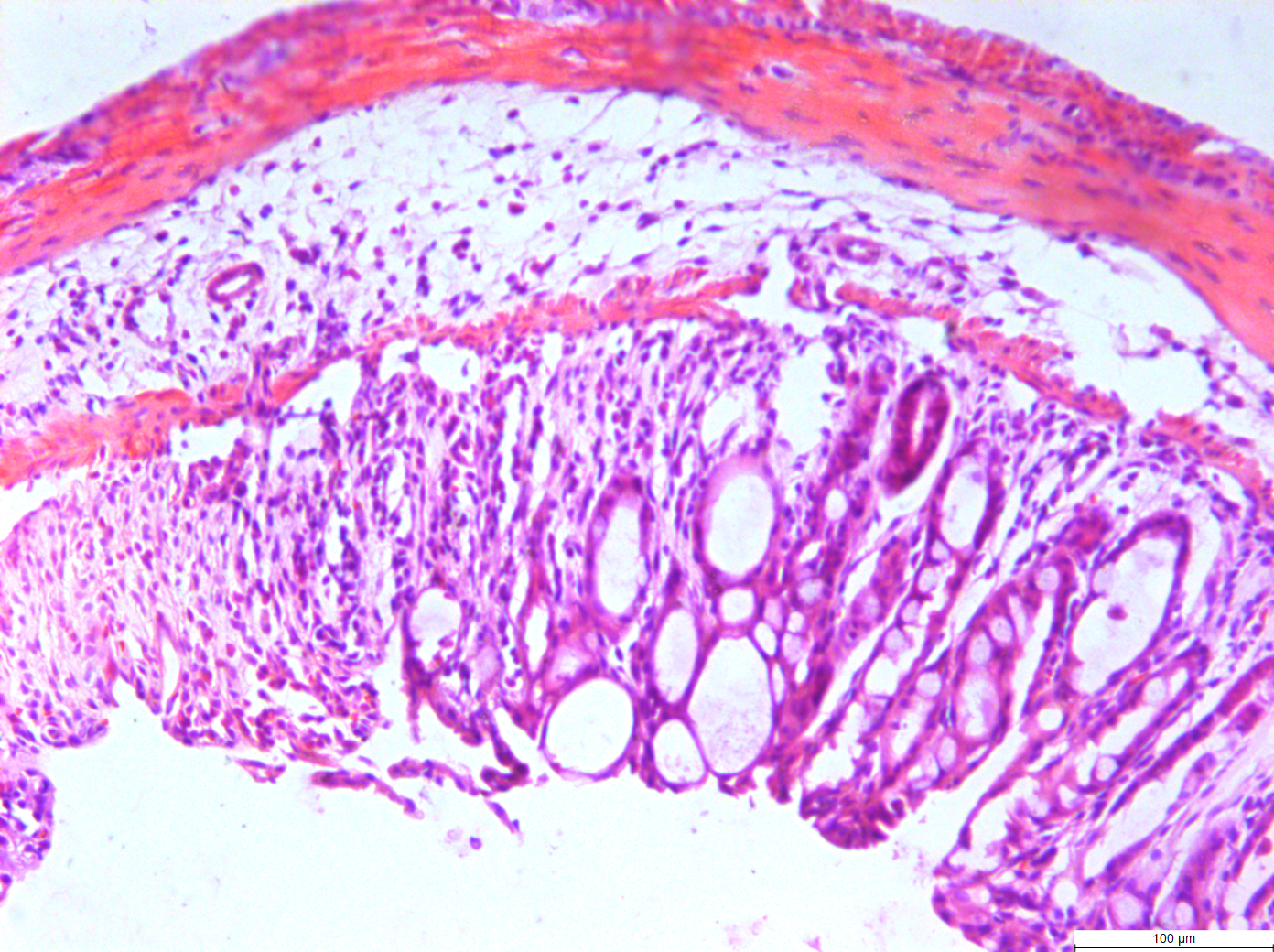

Supplement: S1 Data — (ZIP) [file pone.0291543.s006.zip › HE/DSS+ANINEO-5mg per kg/DSS+ANINEO-5mg per kg 2.tif]

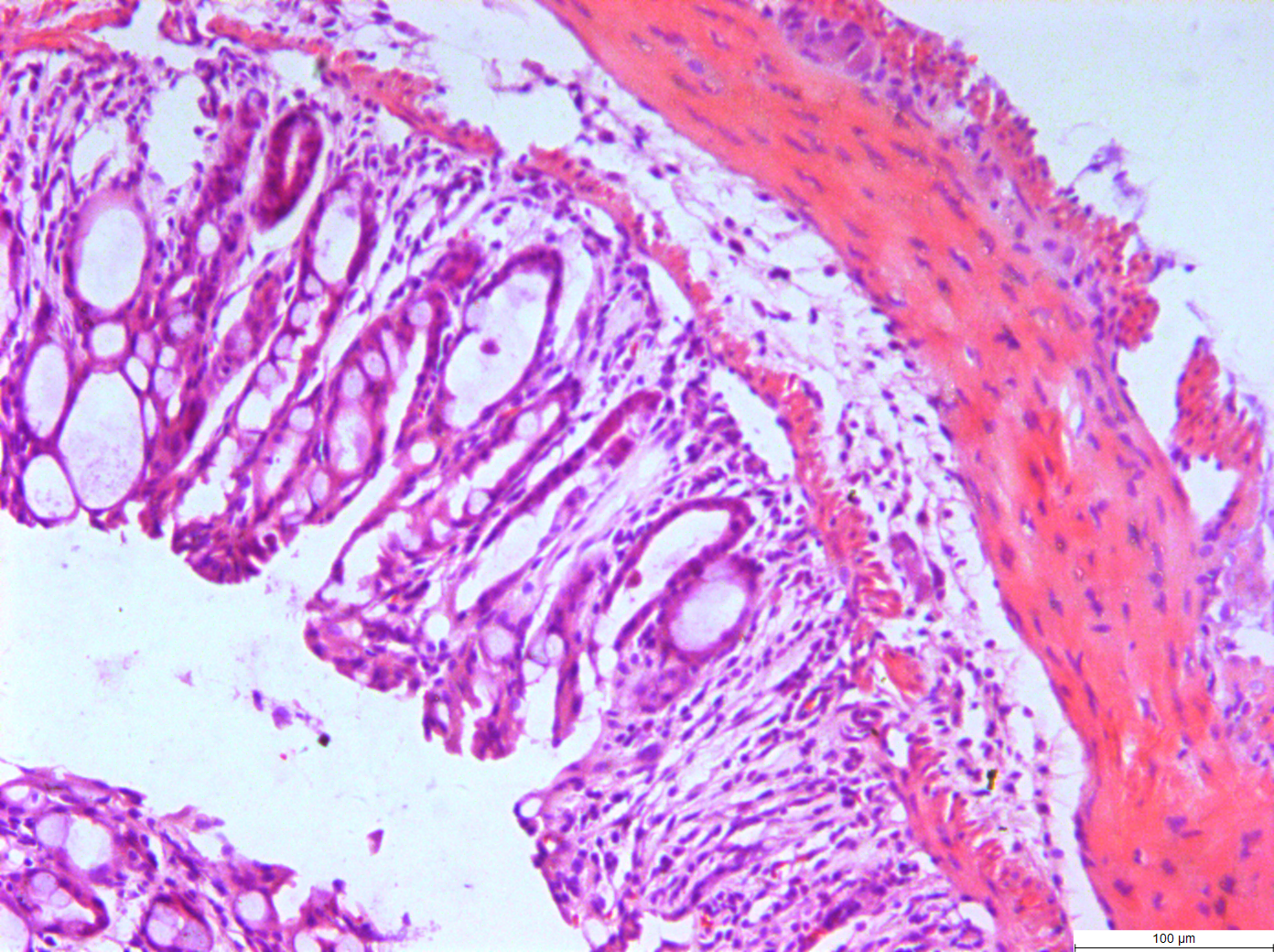

Supplement: S1 Data — (ZIP) [file pone.0291543.s006.zip › HE/DSS+ANINEO-5mg per kg/DSS+ANINEO-5mg per kg 3.tif]

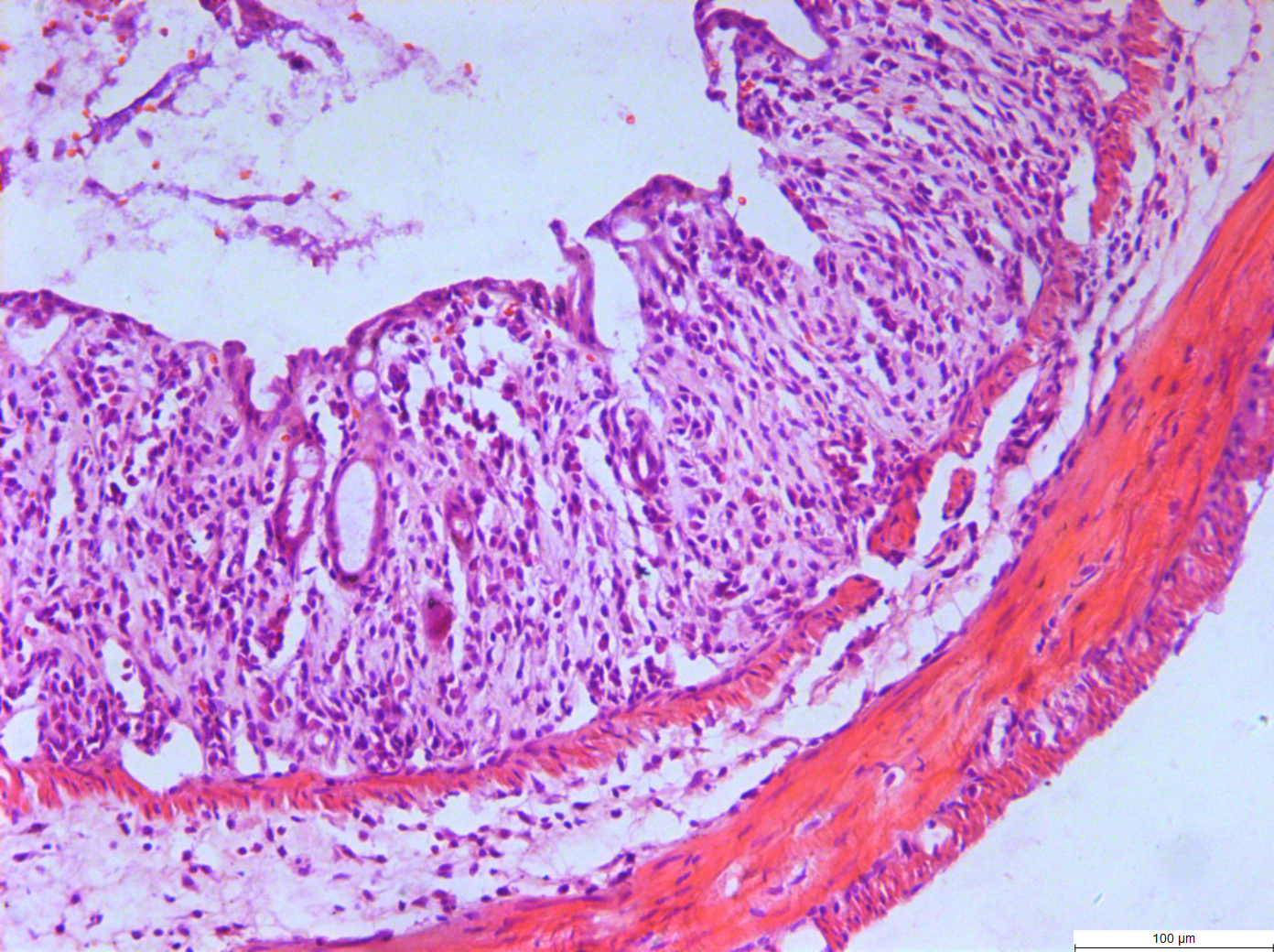

Supplement: S1 Data — (ZIP) [file pone.0291543.s006.zip › HE/DSS+ANINEO-5mg per kg/DSS+ANINEO-5mg per kg 4.tif]

Figure3

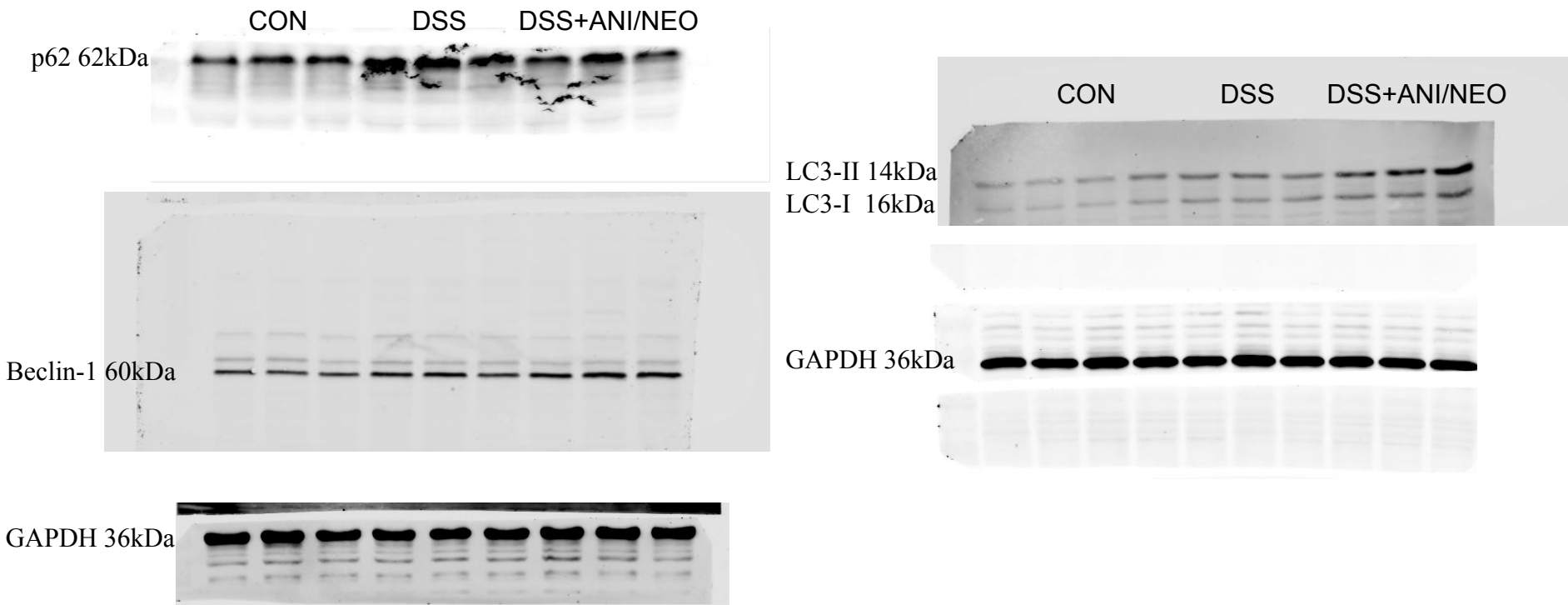

Figure4

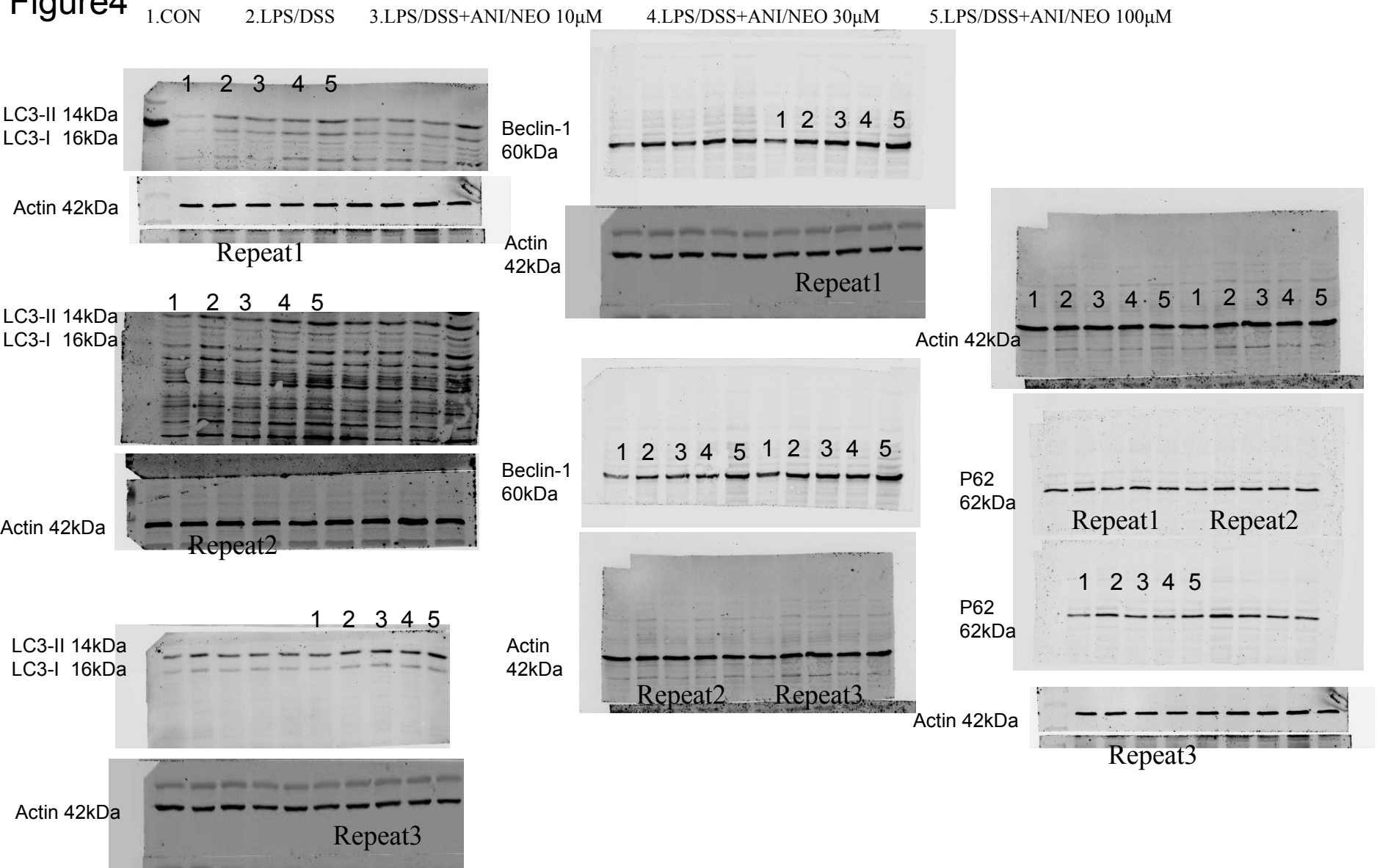

Supplement: S1 Data — (ZIP) [file pone.0291543.s006.zip › original western blot.pdf]

## Slide 1
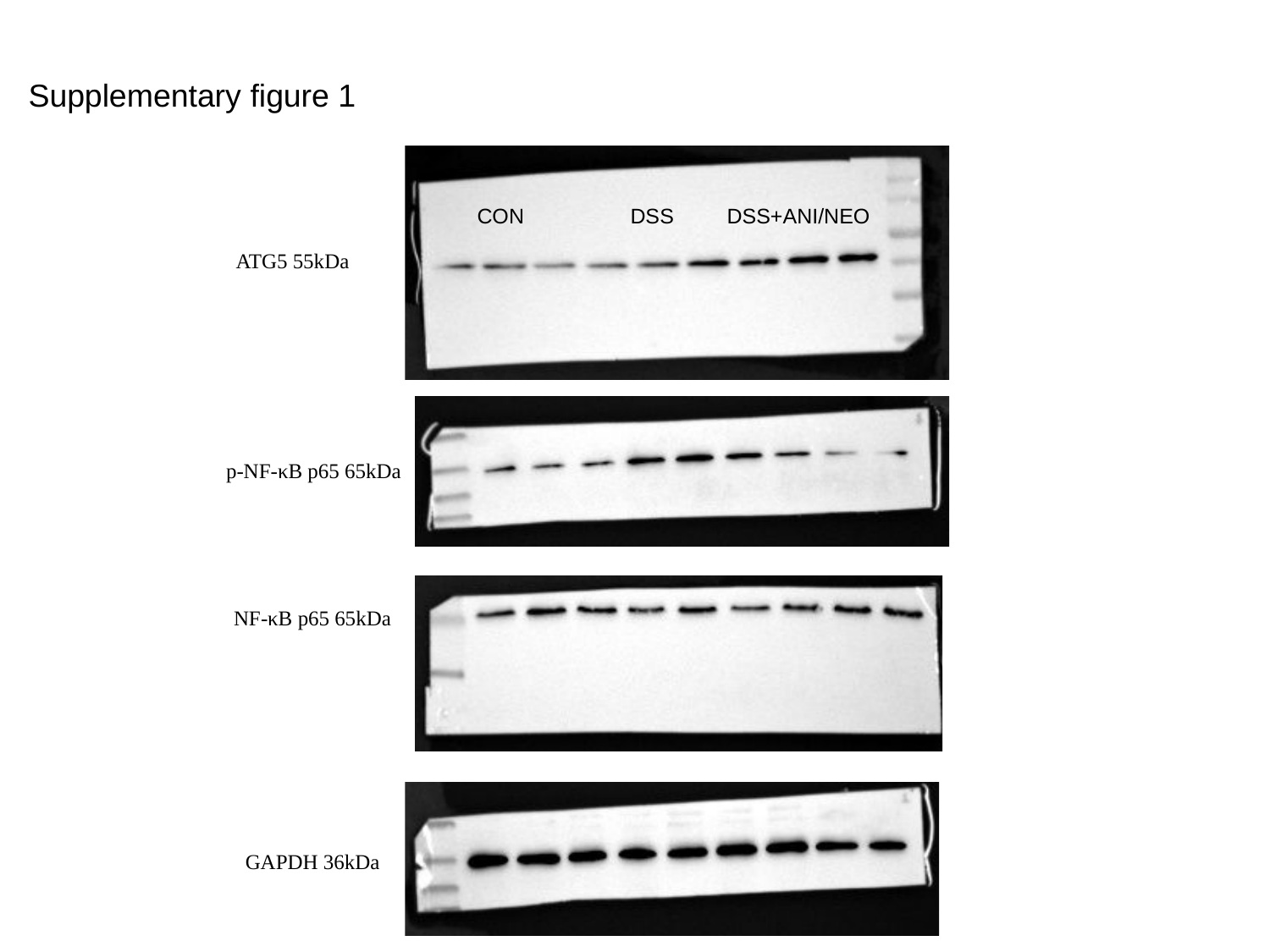

Supplementary figure 1
CON DSS DSS+ANI/NEO
ATG5 55kDa
p-NF-κB p65 65kDa
NF-κB p65 65kDa
GAPDH 36kDa

Supplement: S2 Raw images — (PPTX) [file pone.0291543.s009.pptx]
