# Supplementary material for: Combined administration of anisodamine and neostigmine alleviated colitis by inducing autophagy and inhibiting inflammation
Source: PLoS One. 2024 Feb 14;19(2):e0291543. doi: 10.1371/journal.pone.0291543 (PMC10866466; doi:10.1371/journal.pone.0291543)
Supplement: Attachment — Submitted filename: Response to reviewers.docx [file pone.0291543.s010.docx]

**ITEMIZED RESPOSES TO REVIEWERS’ COMMENTS**

**To Reviewer 2:**

1. The authors did not provide the rationale for using ANI and NEO in intestinal inflammation. Please add a details explanation to the manuscript.

**Re:** Our groups have studied the therapeutic effects of anisodamine and neostigmine for many years, and found out that the anti-inflammatory effect is best when the ratio of anisodamine and neostigmine is at 500:1 in a variety of animal models (septic shock, acute lethal crush syndrome and collagen-induced arthritis, etc.) (doi: 10.1038/aps.2012.26, doi: 10.1038/srep37709, doi: 10.1111/cns.12213). Therefore, we directly used anisodamine and neostigmine combination at this ratio in DSS-induced colitis. From the pharmacodynamic indicators of colitis, the anti-inflammatory effect of this combination is very effective, which is consistent with our previous research.

2. To provide more mechanistic insight, authors can also examine the phosphorylation levels of STAT3 and NFkB in the same region, as these are the central mediators of inflammation.

**Re:** Thank you for your professional suggestion. The phosphorylation level of NF-kB p65 in colon tissue was examined by western blot (S1 figure, Line 233-236, Line 315-320).

3. IL-1B is the main cytokine, which is known to be upregulated up to thousand-fold in DSS treated mice. It would be important to study the main cytokine which exerts the effect after DSS treatment.

**Re:** Thank you for your professional suggestion. The levle of IL-1β in colon tissue was detected by ELISA in S2 figure (Line 230-233).

4. It would be also great to examine the cytokines levels in mice samples treated with MLA as shown in fig 8.

**Re:** Thank you for your professional suggestion. The mRNA levels of INF-γ, TNF-α, IL-6, IL-22 in colon tissue were assessed by RT-PCR (S3 figure, Line 270-274).

**To Reviewer 3:**

1. The authors should highlight what is novel in the current study compared to previous studies (Refs. 10, 32, 33 and 34).

**Re:** Our innovations are as follows: (1) ANI/NEO combination provided protective effect on DSS-induced colitis; (2) The target of ANI/NEO combination to protect colitis is α7nAChR, indicating that it is an a7 receptor agonist which is not reported previously; (3) After activating α7nAChR, ANI/NEO combination inhibited inflammation by promoting the autophagy of intestinal epithelial cells induced by DSS.

2. The authors showed opposing effects of DSS and ANI/NEO on various pro-inflammatory cytokines, while both DSS and ANI/NEO showed similar induction in autophagy markers (LC3-II/LC3I ratio and Beclin 1 levels). It is suggested that ANI/NEO mediates its protective effects by inducing autophagy. However, DSS was also shown to increase LC3-II/LC3I ratio and Beclin 1 levels. Is DSS also protective? How do the authors decipher this aspect?

**Re:** Autophagy is the main intracellular degradation mechanism, through which substances are transported to and degraded in lysosomes. As a dynamic circulatory system, autophagy maintains cell renovation and homeostasis. But excessive autophagy will destroy cell homeostasis and cuase pathological conditions (doi: 10.1080/15548627.2020.1847462, doi: <https://doi.org/10.1016/j.cell.2011.10.026).> The initial augmented autophagy induced by DSS is a compensatory reaction of cells, which is helpful to the maintenance of cell homeostasis ( doi: [10.1080/15548627.2020.1847460](https://doi.org/10.1080%2F15548627.2020.1847460)). However, excessive autophagy caused by continuous stimulation will raise cell apoptosis and aggravate colitis (doi: 10.3389/fphar.2021.697360). The autophagy enhancement after ANI/NEO treatment is only an appearance. Whether it is beneficial or harmful to cells depends on inflammation, tissue damage and other indicators. Altogether the effect of enhanced autophagy on diseases requires further detection of cell function and disease indicators.

3. It will be helpful to assess the protein levels of other autophagy markers such as ATG5 and ATG7 in the colon of DSS colitis mice. This will support the in vitro ATG5 siRNA studies in Caco2 cells.

**Re:** Thank you for your professional suggestion. The protein level of ATG5 in colon tissue was assessed by western blot and was showed in S1 figure (Line 225-227).
