# Supplementary material for: Combined administration of anisodamine and neostigmine alleviated colitis by inducing autophagy and inhibiting inflammation
Source: PLoS One. 2024 Feb 14;19(2):e0291543. doi: 10.1371/journal.pone.0291543 (PMC10866466; doi:10.1371/journal.pone.0291543)
Supplement: Attachment — Submitted filename: Response to reviewers.docx [file pone.0291543.s011.docx]

**Response to Reviewers**

**To Reviewer 4:**

1. Fig. 4: In LPS/ ANI/NEO treated Caco-2 cells, LC3I and II can be blotted in the presence (and absence) of Bafilomycin A to demonstrate the effect of ANI/NEO on autophagic flux.

**Re:** Thanks for your suggestions! We blocked autophagy by ATG5 siRNA before LPS/DSS or LPS/DSS+ANI/NEO and then detected the expression of LC3 using WB. We found that LPS/DSS+ANI/NEO could significantly increase the LC3II/LC3I in the control siRNA group, whereas in ATG5siRNA group, LPS/DSS+ANI/NEO couldn’t increase the expression of LC3Ⅱ/LC3Ⅰ ratio (shown as Figure S4A-B).

2. As authors stated that ANI and NEO combination protects mice from DSS colitis by activating α7nAChR, is there a way to show that ANI/NEO actually activated α7nAChR or MLA inhibited α7nAChR in the mouse model?

**Re:** To explicit the role of α7nAChR in DSS-induced colitis, we compared the therapeutic effect of ANI/NEO on colitis between α7nAChR gene knockout and wild type mice. The findings showed that α7nAChR KO partly abolished the beneficial effects of ANI/NEO on weight loss, DAI, and colon length, which suggests that ANI/NEO at least partially activated α7nAChR (Figure S5A-D).

MLA is a selective antagonist of α7nAChR and has been commonly used to confirm the role of α7nAChR in various disease models (doi: 10.1124/pharmrev.120.000097, doi: 10.1186/s12974-021-02341-6, doi: 10.1161/STROKEAHA.111.639989, doi: 10.1016/j.biopha.2019.109231). Thus, we used MLA to block α7nAChR in this colitis model.

3. Does blocking of α7nAChR affects autophagy in mouse colon?

**Re:** We found that knockout α7nAChR in mouse colon resulted in decreased autophagy, as indicated by decreased LC3Ⅱ/LC3Ⅰ ratio (Figure S5E-F).

4. It will be beneficial to add some information on α7nAChR in the discussion on what type of cells express α7nAChR and its physiological relevance and function, and possible mechanisms underlying its regulation by autophagy.

**Re:** Thank you for your professional suggestion. We have added the concerns in the Discussion in the revised manuscript (Line 355-371).
